# The Functional Role of Hyperpolarization Activated Current (*I*_f_) on Cardiac Pacemaking in Human vs. in the Rabbit Sinoatrial Node: A Simulation and Theoretical Study

**DOI:** 10.3389/fphys.2021.582037

**Published:** 2021-08-19

**Authors:** Xiangyun Bai, Kuanquan Wang, Mark R. Boyett, Jules C. Hancox, Henggui Zhang

**Affiliations:** ^1^Biological Physics Group, Department of Physics and Astronomy, The University of Manchester, Manchester, United Kingdom; ^2^School of Computer Science and Technology, Xi'an University of Posts and Telecommunications, Xi'an, China; ^3^School of Computer Science and Technology, Harbin Institute of Technology, Harbin, China; ^4^Department of Biomedical Sciences, Faculty of Health and Medical Sciences, University of Copenhagen, København, Denmark; ^5^School of Physiology, Pharmacology and Neuroscience, Biomedical Sciences Building, University Walk, Bristol, United Kingdom; ^6^Peng Cheng Laboratory, Shenzhen, China; ^7^Key Laboratory of Medical Electrophysiology of Ministry of Education, Medical Electrophysiological Key Laboratory of Sichuan, Institute of Cardiovascular Research, Southwest Medical University, Luzhou, China

**Keywords:** human and rabbit sinoatrial node, funny current, bradycardic agent (ivabradine), electrophysiological simulation, theoretical analysis

## Abstract

The cardiac hyperpolarization-activated “funny” current (*I*_f_), which contributes to sinoatrial node (SAN) pacemaking, has a more negative half-maximal activation voltage and smaller fully-activated macroscopic conductance in human than in rabbit SAN cells. The consequences of these differences for the relative roles of *I*_f_ in the two species, and for their responses to the specific bradycardic agent ivabradine at clinical doses have not been systematically explored. This study aims to address these issues, through incorporating rabbit and human *I*_f_ formulations developed by Fabbri et al. into the Severi et al. model of rabbit SAN cells. A theory was developed to correlate the effect of *I*_f_ reduction with the total inward depolarising current (*I*_total_) during diastolic depolarization. Replacing the rabbit *I*_f_ formulation with the human one increased the pacemaking cycle length (CL) from 355 to 1,139 ms. With up to 20% *I*_f_ reduction (a level close to the inhibition of *I*_f_ by ivabradine at clinical concentrations), a modest increase (~5%) in the pacemaking CL was observed with the rabbit *I*_f_ formulation; however, the effect was doubled (~12.4%) for the human *I*_f_ formulation, even though the latter has smaller *I*_f_ density. When the action of acetylcholine (ACh, 0.1 nM) was considered, a 20% *I*_f_ reduction markedly increased the pacemaking CL by 37.5% (~27.3% reduction in the pacing rate), which is similar to the ivabradine effect at clinical concentrations. Theoretical analysis showed that the resultant increase of the pacemaking CL is inversely proportional to the magnitude of *I*_total_ during diastolic depolarization phase: a smaller *I*_f_ in the model resulted in a smaller *I*_total_ amplitude, resulting in a slower pacemaking rate; and the same reduction in *I*_f_ resulted in a more significant change of CL in the cell model with a smaller *I*_total_. This explained the mechanism by which a low dose of ivabradine slows pacemaking rate more in humans than in the rabbit. Similar results were seen in the Fabbri et al. model of human SAN cells, suggesting our observations are model-independent. Collectively, the results of study explain why low dose ivabradine at clinically relevant concentrations acts as an effective bradycardic agent in modulating human SAN pacemaking.

## Introduction

The pacemaker activity of sinoatrial node (SAN) cells in the mammalian heart arises from the integrated action of multiple sarcolemmal ionic channel currents and the interaction between the intracellular calcium handling and sarcolemmal electrogenic processes (Irisawa et al., 1993; Mangoni and Nargeot, 2008; Lakatta et al., 2010). The hyperpolarization-activated “funny” current, *I*_f_, present in the SAN and other regions of the cardiac conduction system (Boyett, 2009; Difrancesco, 2010), is produced by the hyperpolarization-activated cyclic nucleotide gated (HCN) channel isoforms (of which there are four: HCN1-4), each of which is comprised of six transmembrane domains with four subunits combining to produce functional tetrameric channels, as occurs for voltage-gated potassium channels (Bois et al., 2007; Difrancesco, 2010). Previous studies of the rabbit SAN have shown that although HCN isoforms 1, 2, and 4 are all expressed in the heart, HCN4 is the most abundant in the SAN and the *I*_f_ density within SAN sub-regions correlates strongly with HCN4 expression levels (Thollon et al., 2007; Brioschi et al., 2009). The unique features of HCN channels lie in the fact that they are activated not on depolarization but on hyperpolarization of cell membrane potential (to voltages negative to ~−40 to ~−50 mV) (Hagiwara and Irisawa, 1989; Accili et al., 1997; Baruscotti et al., 2005) and are permeable to both Na^+^ and K^+^ ions, with an approximate reversal potential of −30 mV (Van Ginneken and Giles, 1991; Verkerk et al., 2009a). Upon hyperpolarization, HCN channels generate an inward current over the pacemaking potential range which, together with the current generated by other electrogenic processes of the intracellular calcium handling (i.e., the Ca^2+^ clock), contributes to the genesis of intrinsic pacemaker activity of the SAN (Lakatta and Difrancesco, 2009). HCN channels are also modulated by adrenergic agonists *via* cAMP (Bucchi et al., 2003; Craven and Zagotta, 2006).

*I*_f_ channels are also present in the human SAN. It has been shown that HCN4 is strongly expressed in the human SAN, with the measured mRNA level of other isoforms accounting for <16% (with HCN3 being negligible, accounting for only 0.5%) of the total mRNA measurement (Chandler et al., 2009). Though the HCN expression in the human SAN is similar to that in the rabbit, properties and kinetics of *I*_f_ are clearly different. It has been shown the fully-activated *I*_f_ conductance in the human is about 3–4 times smaller than that in the rabbit (Verkerk et al., 2007a). Additionally, *I*_f_ in human SAN cells has a more negative half-maximal activation voltage, and a greater time constant of deactivation/activation process which is also negatively shifted (Verkerk et al., 2007a). With such a marked species differences in *I*_f_ conductance and kinetics between the rabbit and human, a question arises as to whether a smaller *I*_f_ in the human SAN cells plays the same important role in regulating cardiac pacemaking activity as in the rabbit SAN?

Although *I*_f_ in human SAN cells is much smaller than those in other mammals, it may play a comparable role to that in the rabbit in modulating cardiac pacemaking. In their study, (Verkerk et al., 2007b) observed about a 26% increase in pacemaking cycle length in human SAN cells on complete block of *I*_f_ by using 2 mM Cs^+^, which is close to that seen in the rabbit (Verkerk and Wilders, 2010; Fabbri et al., 2017). Pharmacological targeting of *I*_f_ by ivabradine has also shown the clinical value of *I*_f_ in controlling the heart rate in patients who need heart rate control in conditions of coronary artery disease (CAD) (Tardif et al., 2005; Camici et al., 2016; Niccoli et al., 2017) and heart failure (HF) (Bohm et al., 2015; Yancy et al., 2016). In both conditions, slowing down the heart rate by ivabradine increases the diastolic interval, thereby reducing the metabolic load on the working myocardium; this reduces the risks of ischemia of the heart leading to reduced risk of sudden death (Niccoli et al., 2017).

Although inhibition of *I*_f_ by ivabradine provides an efficient pharmacological control of heart rate in the clinic, it is still unclear how the clinical concentration range of ivabradine [about 20–140 nM (Choi et al., 2013; Jiang et al., 2013); blocking *I*_f_ < 20% (Bois et al., 1996; Bucchi et al., 2002)] can produce a significant effect in reducing human heart rate. In pre-clinical animal model studies, ivabradine has been reported to inhibit *I*_f_ in SAN cells with a half-maximal inhibitory concentration of 1.5–2.8 μM (Bois et al., 1996; Bucchi et al., 2002), and recombinant HCN4 channels with an IC_50_ values between 0.5 and 2.0 μM (Bucchi et al., 2002, 2006, 2013). In rabbit SAN cells, 1 μM ivabradine has been observed to reduce the pacemaking rate by 12.3% (Thollon et al., 1994), whilst about 16.2 and 23.8% of heart rate reduction at 3 μM have been seen (Thollon et al., 1994; Bucchi et al., 2007).

When ivabradine was administered intravenously (0.2 mg kg^−1^) to patients with normal baseline electrophysiology, mean heart rate reductions of 12.9 and 14.1 beats min^−1^ (at 0.5 and 1 h respectively following administration) were observed (Camm and Lau, 2003). The drug is usually administered orally, however, and after repeated oral dosing at 5 mg, mean maximal plasma (C_max_) levels of 11–16 ng ml^−1^ (23.5–34.1 nM) have been measured, whilst for repeated dosing at 10 mg, mean C_max_ levels of 29–42 ng ml^−1^ (61.8–89.6 nM) have been seen (Choi et al., 2013; Jiang et al., 2013). Repeated dosing with a high ivabradine concentration of 20 mg has been associated with a C_max_ of 137 nM (Jiang et al., 2013). On the basis of the pre-clinically observed concentration-dependence for *I*_f_ inhibition, comparatively low levels of *I*_f_ block might be expected at such plasma levels (Thollon et al., 1994; Bucchi et al., 2007). However, clinical concentrations of ivabradine produce about 18–20% reduction in human heart rate (Camm and Lau, 2003; Doesch et al., 2007).

Previous animal model studies (Difrancesco, 1991, 2010) have found that inhibition of *I*_f_ by ivabradine slowed down the spontaneous firing rate of the rabbit SAN cell in a use-dependent manner (Bois et al., 1996), and about 15% reduction of the pacemaking rate was observed at a concentration of 3 μM, which produced about 60% *I*_f_ reduction at membrane potential of −92 mV, and about 41% *I*_f_ reduction at physiologically relevant membrane potentials (~60 mV) (Yaniv et al., 2012). Numerically, in a recent in silico exploration of the role of *I*_f_ in SAN pacemaking using a rabbit SAN model, the effect of ivabradine was simulated through implementing a 66% reduction of *I*_f_ (mimicking an experimentally reported effect of 3 μM ivabradine), leading to a 22% reduction in spontaneous rate (Severi et al., 2012). However, due to the non-linear concentration-dependent action of ivabradine on *I*_f_, it is hard (if not impossible) to derive the effect of a low concentration of ivabradine from that of a high concentration on modulating cardiac pacemaking rate. To date, therefore, the effect of blocking *I*_f_ at the level of *I*_f_ reduction at clinical plasma levels of ivabradine (<20%) on pacemaking rate of the rabbit SAN has not been elucidated, as is how such concentration can produce a marked effect on the human SAN, in which *I*_f_ is much smaller than that in the rabbit SAN. Most importantly, it is unclear either how clinical concentrations of ivabradine affect cardiac pacemaking activity *in vivo* as compared to those predicted by single cell experiments *in vitro*, in which vagal tone modulation of cardiac pacemaking is missing. The aim of the present study was therefore to investigate through simulations and theoretical analysis the effect of *I*_f_ reduction over a wide range of values on cardiac pacemaking activity in the presence and absence of vagal tone modulation of cardiac pacemaking activity by acetylcholine.

## Methods

### SAN Cell Model and *I*_f_ Formulations

In this study, the contemporary model of rabbit SAN cells developed by Severi et al. (2012) was used as a basal model. The model was chosen as it represents the most updated progress in the model development of rabbit SAN cells, in particular it incorporates an updated *I*_f_ formulation based on recently available experimental data (Altomare et al., 2003; Barbuti et al., 2007). The basal model code was downloadable from cellML at the following URL: https://models.physiomeproject.org/e/139; and the source codes used for this study are available on request to: henggui.zhang@manchester.ac.uk. In brief, the dynamics of the membrane action potential the SAN cell were modeled as:

(1) $$\begin{array}{l} \frac{dV}{dt}=-\frac{i_{total}}{C_{m}} \end{array}$$

$$\begin{array}{r} i_{total}=I_{CaL}+I_{CaT}+I_{Kr}+I_{Ks}+I_{sus}+\\ I_{to}+I_{NaK}+I_{NaCa}+I_{Na}+I_{f} \end{array}$$

Where V is the membrane potential, C_m_ the membrane capacitance, *t* the time, *I*_total_ the total membrane current generated by potassium (*I*_Kr_, *I*_Ks_, *I*_sus_, *I*_to_), calcium (*I*_CaL_, *I*_CaT_), sodium (*I*_Na_), Na^+^-K^+^ pump (*I*_NaK_), Na^+^-Ca^2+^ exchanger (*I*_NaCa_), and funny (*I*_f_) channels. More details of the basal model are documented in the study of Severi et al. (2012).

Previous experimental studies have shown some distinctive differences in the maximal macroscopic conductance, the steady-state activation curve and the time constant of the channel activation of *I*_*f*_ between the rabbit and the human SAN cells (Difrancesco et al., 1989; Altomare et al., 2003; Barbuti et al., 2007; Verkerk et al., 2007b). In order to take into consideration the reported species difference in *I*_f_ properties, in our simulations we implemented two different sets of *I*_f_ formulations, one is the original model formulations developed by Severi et al. (2012) (rabbit-like formulation), and the other is Fabbri et al. formulation (Fabbri et al., 2017) based on the human *I*_f_ data (Verkerk et al., 2007b) (human-like formulation), which takes the form:

$$\begin{array}{l} I_{\text{f}}=I_{\text{f,Na}}+I_{\text{f,K}} \end{array}$$

$$\begin{array}{l} I_{\text{f,Na}}=g_{\text{f,N}a}\cdot y\cdot \left(V-E_{\text{Na}}\right) \end{array}$$

$$\begin{array}{l} I_{\text{f,K}}=g_{\text{f,K}}\cdot y\cdot \left(V-E_{\text{K}}\right) \end{array}$$

$$\begin{array}{l} \tau_{y}=\frac{1}{(0.36\cdot \left(V+148.8\right)/\left(e^{0.066\cdot \left(V+148.8\right)}-1\right)+0.1\cdot \left(V+87.3\right)/\left(1-e^{-0.21\cdot \left(V+87.3\right)}\right)}-0.054 \end{array}$$

$$\begin{array}{r} y_{\infty }=0.01329+0.99921/\left(1+e^{\left(V+97.134\right)/8.1752}\right),\\ \text{if V}<-80\text{mV} \end{array}$$

$$\begin{array}{l} y_{\infty }=0.0002501\cdot e^{\left(-V/12.861\right)},\text{if V}\geq -80\text{mV} \end{array}$$

$$\begin{array}{l} \frac{dy}{dt}=\frac{y_{\infty }-y}{\tau_{y}} \end{array}$$

where *I*_f,Na_ and *I*_f,K_ are Na^+^ and K^+^ components of *I*_f_, *g*_f,Na_ (0.00268 μS) and *g*_f,K_ (0.00159 μS) conductance of *I*_f,Na_ and *I*_f,K_. *E*_Na_ and *E*_K_ the equilibrium potential for Na^+^ and K^+^. *y*_∞_ is the steady state activation variable, τ_y_ the time constant of activation variable (*y*).

**Figure 1:**
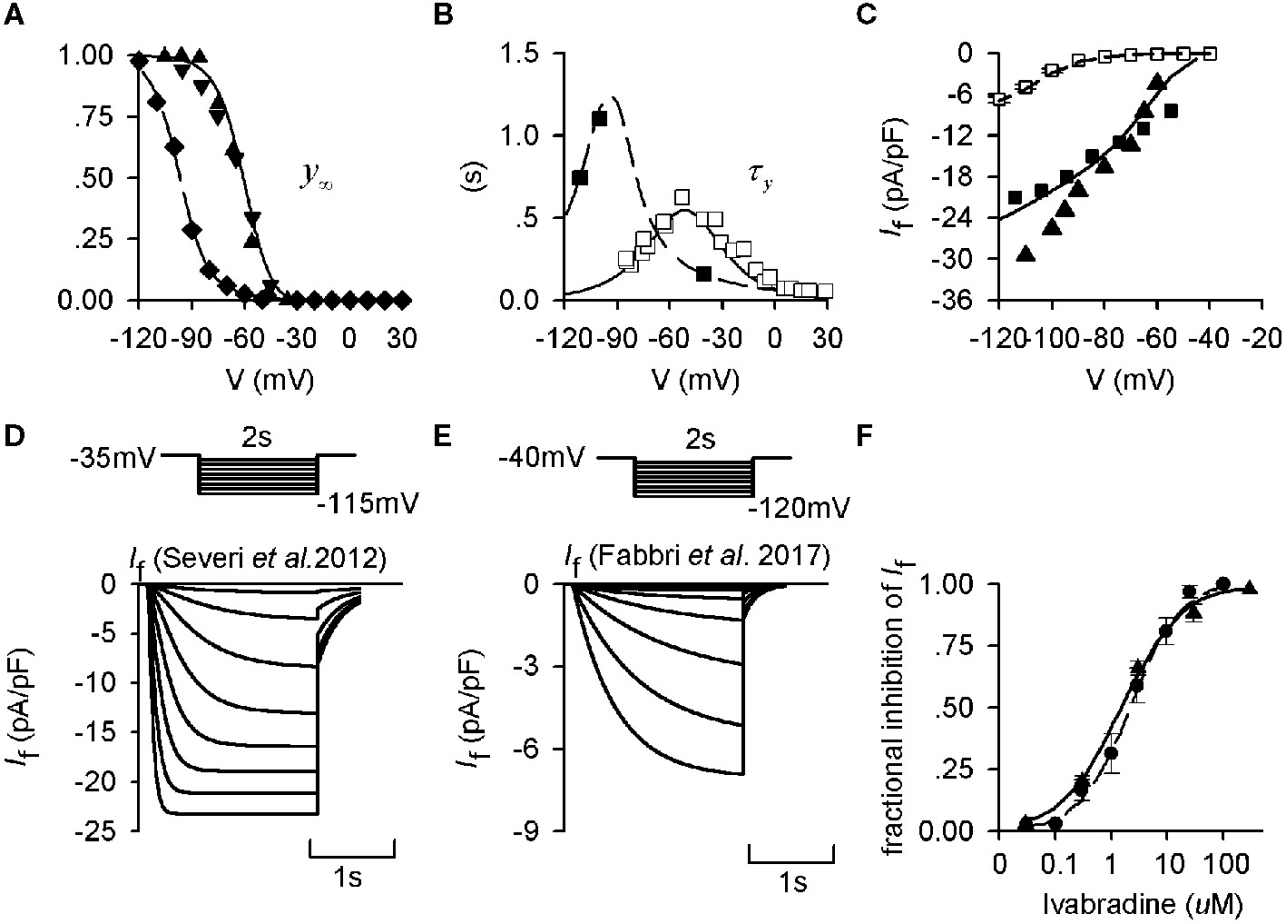
Kinetics of *I*_f_ from experimental and mathematical modeling data. **(A)** Steady-state activation curves in rabbit and human and SAN cells, which were used to fit *I*_f_ formulations for the Severi et al. (2012) rabbit (solid lines) and the modified Fabbri et al. (2017) human (dashed lines) models. Experimental data from Altomare et al. (2003) (▴), Barbuti et al. (2007) (▾), and Verkerk et al. (2007b) (♦) are also shown. **(B)** Corresponding activation time constant of *I*_f_ for human and rabbit SAN cells. Experimental data from Difrancesco et al. (1989) (□) and Verkerk et al. (2007b) (■) are also shown. **(C)** Computed *I*-*V* relationship for the human- (dashed line) and rabbit-like (solid line) models, which are compared with experimental data from the study of Verkerk et al. (2007b) (□) for the human SAN cells, Zaza et al. (1996) (■) and Goethals et al. (1993) (▴) for the rabbit SAN cells. **(D,E)** Voltage-clamp protocol and simulated time traces of *I*_f_ during voltage-clamp for the rabbit and human *I*_f_ model. **(F)** Dose-dependent effect of ivabradine on *I*_f_ in rabbit SAN cells. Experimental data of Bois et al. (1996) (•) and Bucchi et al. (2002) (▴) were based to develop the concentration-response curves as shown by the solid line and dashed line respectively.

To determine and validate the parameters in the equations of human-like and rabbit-like *I*_f_ formulations, the equations for the steady-state activation curves (Figure 1A), and the equation for the activation time constant (Figure 1B) were fitted to experimental data obtained from human and rabbit SAN cells respectively (Difrancesco et al., 1989; Altomare et al., 2003; Barbuti et al., 2007; Verkerk et al., 2007b). The developed *I*_f_ formulations were validated by their ability to reproduce experimental I-V relationship data (Figure 1C) obtained by running a series of voltage-clamp commands (Figure 1D) for both the human-like and rabbit-like formulations. Figure 1 shows clearly that *I*_f_ in the human SAN has a more negative half-maximal activation voltage (Figure 1A), a greater activation time constant (i.e., slower activation process (Figure 1B) and a smaller current density (Figure 1C) than that in the rabbit SAN.

### Simulating the Effects of *I*_f_ Blockade

It has been shown that ivabradine blocks *I*_f_ without affecting channel kinetics, with block leading to a constant level of *I*_f_ reduction after a period of transition (Bucchi et al., 2002, 2013). Therefore, in this study, we implemented a pore-block theory (Yuan et al., 2015) to simulate the steady-state effect of *I*_f_ blocking by ivabradine over a wide concentration range by reducing its conductance by a factor *k* (*k*∈ (0,1), mimicking 0–100% (Bucchi et al., 2013) of *I*_f_ reduction. With varying levels of *I*_f_ inhibition, by the pore-block theory the *I*_f_ conductance became:

$$\begin{array}{l} \{\begin{array}{l} g_{\text{fNa}}^{\prime }=\left(1-k\right)\cdot g_{\text{fNa}}\\ g_{\text{fK}}^{\prime }=\left(1-k\right)\cdot g_{\text{fK}} \end{array} \end{array}$$

### Simulating the Effect of Acetylcholine (ACh)

*In vivo*, ACh released from vagal activity slows down the spontaneous pacing rate of the SAN mainly by inhibiting *I*_f_ and *I*_*CaL*_ (Boyett et al., 1995), and activating acetylcholine-dependent K^+^ current (*I*_KACh_) (Voigt et al., 2014). Vagal activity may also play a significant role in slowing down the pacing rate when ivabradine blocks *I*_f_. To test the combined action of ivabradine and ACh, we simulated the ACh effect on SAN spontaneous APs, based on the formulations of Severi et al. (2012) for *I*_f_ and *I*_CaL_ inhibition, as well as *I*_KACh_ activation. In simulations, the values of g_K,ACh_ used were the same as those used in the Severi et al. (2012) and Fabbri et al. models (Fabbri et al., 2017) for the human-like model (Severi model with human-like *I*_f_) ((Fabbri et al., 2017) and the rabbit-like model (i.e., the Fabbri et al. model with rabbit-like *I*_f_; see details in the Supplementary Material) (Severi et al., 2012). Effects of ACh on pacemaking APs were qualitatively analyzed and compared with the implementation of rabbit-like and human-like *I*_f_ formulation, as well as *I*_f_ blocking. Details of the *I*_KACh_ formulation were listed in the Supplementary Material.

### Study of Model-Dependence

In order to test the model-dependence of results, simulations were also conducted in the Fabbri et al. model of the human SAN cell (Fabbri et al., 2017), the source code of which was downloadable from cellML at the following URL: https://models.physiomeproject.org/e/568?searchterm=human++si. In this case, the Fabbri et al. model with the human-like *I*_f_ formulation was taken as the basal model, which was then modified by replacing the *I*_f_ formulation by the rabbit-like one.

Although simulated action potentials from the original Fabbri et al. (2017) model closely match experiment data of AP properties and calcium transient of human SAN cells, some modification was necessary as most of the ionic currents in the model (except *I*_f_, *I*_Kr_, and *I*_Ks_) were based on rabbit SAN cell model, densities of which were modified by automatic optimization to match simulated action potential characteristics to experimental data. Such automatic optimization of model parameters may deviate from physiological relevance, resulting in some limitations. For example, a full block of *I*_CaT_ in the model abolished the pacemaking action potential. Though there are no direct experimental data from human SAN cells to validate the simulation result, data from rabbit sinoatrial node (Hagiwara et al., 1988; Takeda et al., 2004) and human patients (Madle et al., 2001) suggested a more modest change in the pacemaking cycle length when *I*_CaT_ was blocked. Therefore, we updated the model to address some of the limitations. Details about model updating and validations are presented in Supplementary Figure 1. Using the updated model, simulations in the Serveri et al. model were repeated in the Fabbri et al. model, and results are included in the Supplementary Material to support the major conclusion of the study.

### Numerical Scheme

A fourth-order Runge-Kutta-Merson numerical integration method was used to solve the ordinary differential equations of the model. The time step was 5 × 10^−6^ s, which gives a stable solution of the equations and maintains the accuracy of the computation of membrane current and potential. In simulations, action potentials after the 20th one were recorded for analysis. For solving the Severi et al. model with the human *I*_f_ formulation, a set of initial values were used, which were taken from the recorded state variables when the model reached its steady state (see details about the initial values for solving the model in the Supplementary Material). This allows the secondary effect of different *I*_f_ formulations to other channel variables of the models to be considered in the simulations.

### Theoretical Analysis

Theoretical analysis of the effect of *I*_f_ block on altered cardiac pacemaking cycle length (CL) was conducted following a similar approach as implemented in previous studies (Rocchetti et al., 2000; Zaza and Lombardi, 2001; Monfredi et al., 2014; Winter and Shattock, 2016; Zaza, 2016).

**Figure 2:**
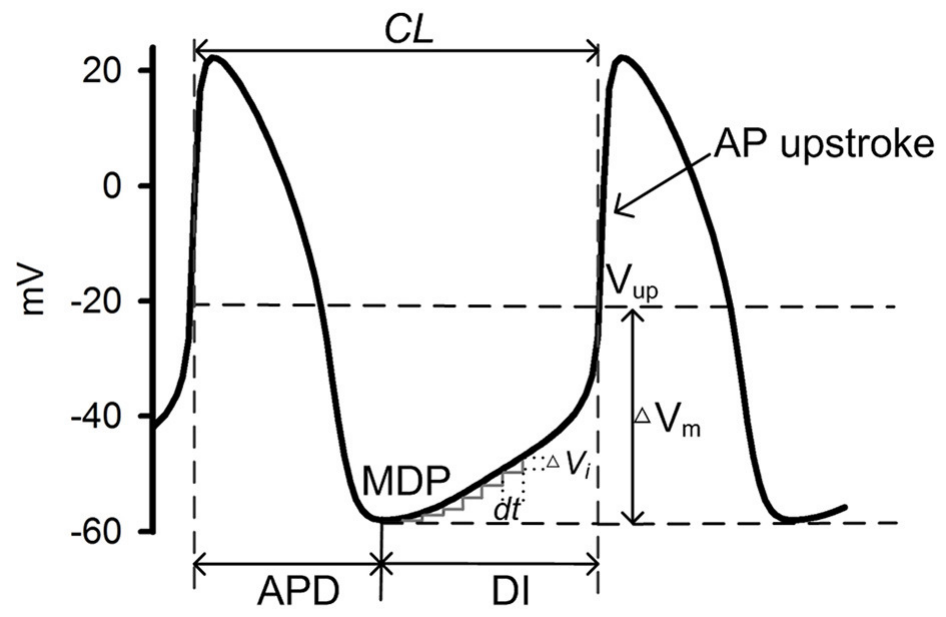
Schematic illustration of a cycle of SAN cell action potentials. During one cycle, the time interval between two consecutive action potentials measures the intrinsic pacemaking CL, which can be considered as a sum of the AP duration (APD) and the diastolic interval (DI) which starts from the maximum diastolic potential (MDP) to the beginning of AP upstroke [V_up_ (Fabbri et al., 2017)]. ΔV_m_ denotes the voltage difference between the MPD and the V_up_. During the DI, multiple time intervals (d*t*) were needed for the membrane potential to transit from the MDP to the V_up_, during each of which a ΔV_i_ is generated.

Figure 2 shows a schematic illustration of a cycle of action potentials of SAN cells. During the time course of the action potential, the voltage difference between the MDP and the voltage at the beginning of AP upstroke (V_up_) (ΔV_m_) can be discretized as many small steps by ΔV_i_, each taking a time period DI_i_ to complete. Here dV_i_/d*t* represents the local diastolic depolarization rate (DDR). During the diastolic depolarization phase, with small time interval (*d*t), |dV_i_/d*t*| can be approximately considered as a constant and denoted by |dV/d*t*|. And the total diastolic interval (DI) can be expressed as:

(2) $$\begin{array}{l} \text{DI}=\sum \text{D}{\text{I}}_{i}=\sum \frac{\Delta {\text{V}}_{i}}{\left|\text{d}{\text{V}}_{i}/\text{d}t\right|}=\frac{\sum \Delta {\text{V}}_{i}}{\left|\text{dV}/\text{d}t\right|}=\frac{\Delta {\text{V}}_{m}}{\left|\text{dV}/\text{d}t\right|} \end{array}$$

Considering Equation (2), the pacemaking cycle length (CL) can be denoted as:

$$\begin{array}{l} \text{CL}=\text{APD}+\text{DI}=\text{APD}+\frac{\Delta {\text{V}}_{m}}{\left|\text{dV}/\text{d}t\right|}=\text{APD}+\frac{{\text{C}}_{m}\Delta {\text{V}}_{m}}{\left|I_{total}\right|} \end{array}$$

Where *I*_total_ denotes total membrane currents during the diastolic depolarization phase.

In response to *I*_f_ block, a new total ion channel current during the diastolic depolarization phase *I'*_total_ is generated, which takes the form

$$\begin{array}{l} I_{total}^{\prime }=I_{total}-\Delta I \end{array}$$

Δ*I* is the change of *I*_total_ caused by *I*_f_ reduction. This produces a new pacing cycle length (CL′), which can be represented as:

$$\begin{array}{l} \text{C}{\text{L}}^{\prime }={\text{APD}}^{\prime }+\frac{{\text{C}}_{m}\Delta {\text{V}}_{m}}{\left|I_{total}^{\prime }\right|}=\text{APD}+\frac{C_{m}\Delta {\text{V}}_{m}}{\left|I_{total}-\Delta I\right|} \end{array}$$

where APD' is the new action potential duration in response to *I*_f_ blockade. As a small *I*_f_ block in response to a low dose of ivabradine mainly affects the diastolic depolarization phase and has little or no impact on the AP duration and the MDP, APD′ is approximately equal to APD. Therefore, the increased cycle length (ΔCL*)* can be represented as

$$\begin{array}{l} \Delta \text{CL}=\text{C}{\text{L}}^{\prime }-\text{CL}={\text{C}}_{m}\Delta {\text{V}}_{m}\left(\frac{1}{\left|I_{total}-\Delta I\right|}-\frac{1}{\left|I_{total}\right|}\right)\\ \;={\text{C}}_{m}\Delta {\text{V}}_{m}\frac{\left|\Delta I\right|}{\left|I_{total}|\cdot |I_{total}-\Delta I\right|} \end{array}$$

Then the relative change of the pacing cycle length is:

$$\begin{array}{l} \frac{\Delta \text{CL}}{\text{CL}}=\frac{{\text{C}}_{m}\Delta {\text{V}}_{m}}{\left(\text{APD}+\frac{{\text{C}}_{m}\Delta {\text{V}}_{m}}{\left|I_{total}\right|}\right)}\cdot \frac{1}{\left|I_{total}\right|}\cdot \frac{\left|\Delta I\right|}{\left|I_{total}-\Delta I\right|} \end{array}$$

By denoting *p* as *I*_f_ current block potency (*p* = |Δ*I*/*I*_f_|), and *x* as the proportion of *I*_f_ to *I*_total_ during the diastolic phase (*x* = |*I*_f_/*I*_total_|), then we have:

$$\begin{array}{l} \frac{\Delta \text{CL}}{\text{CL}}=\frac{{\text{C}}_{m}\Delta {\text{V}}_{m}}{\left(\text{APD}+\frac{{\text{C}}_{m}\Delta {\text{V}}_{m}}{\left|I_{total}\right|}\right)}\cdot \frac{1}{\left|I_{total}\right|}\cdot \frac{\left|\Delta I/I_{f}\right|}{\left|I_{total}/I_{f}-\Delta I/I_{f}\right|}\\ \;=\frac{p}{\left(\frac{\text{APD}}{{\text{C}}_{m}\Delta {\text{V}}_{m}}|I_{total}|+1\right)}\cdot \frac{1}{\left|\frac{1}{x}-p\right|} \end{array}$$

By setting

$$\begin{array}{l} {\text{C}}_{1}=\frac{\text{APD}}{{\text{C}}_{m}\Delta {\text{V}}_{m}} \end{array}$$

where C_1_ (C_1_ > 0) can be considered as a constant during the diastolic depolarization phase with a fixed level of *I*_f_ reduction (i.e., *p* is fixed), which has no significant effect on the difference between MDP and the V_up_ (i.e., Δ V_m_), then we have:

(3) $$\begin{array}{l} \frac{\Delta \text{CL}}{\text{CL}}=\frac{p}{\left({\text{C}}_{1}|I_{total}|+1\right)}\cdot \frac{1}{\left|\frac{1}{x}-p\right|}\propto {\text{C}}_{2}\cdot \frac{1}{\left|I_{total}\right|}\cdot \frac{1}{\left|\frac{1}{x}-p\right|} \end{array}$$

Where C_2_ also can be seen as a constant related to C_1_ and *p*, also with a fixed level of *I*_f_ reduction.

With *I*_f_ block, the resultant relative change of the CL predicted by Equation (3) is inversely proportional to the amplitude of *I*_total_ during the diastolic depolarization phase, which determines the intrinsic CL of the pacemaking action potential. It is also related to the level of *I*_f_ reduction and the ratio between *I*_f_ and *I*_total_. With a small level of *I*_f_ block, the resultant relative change of CL is greater for a smaller *I*_total_ (i.e., greater when the CL is larger or the heart rate is lower).

## Results

### Simulation Results

**Figure 3:**
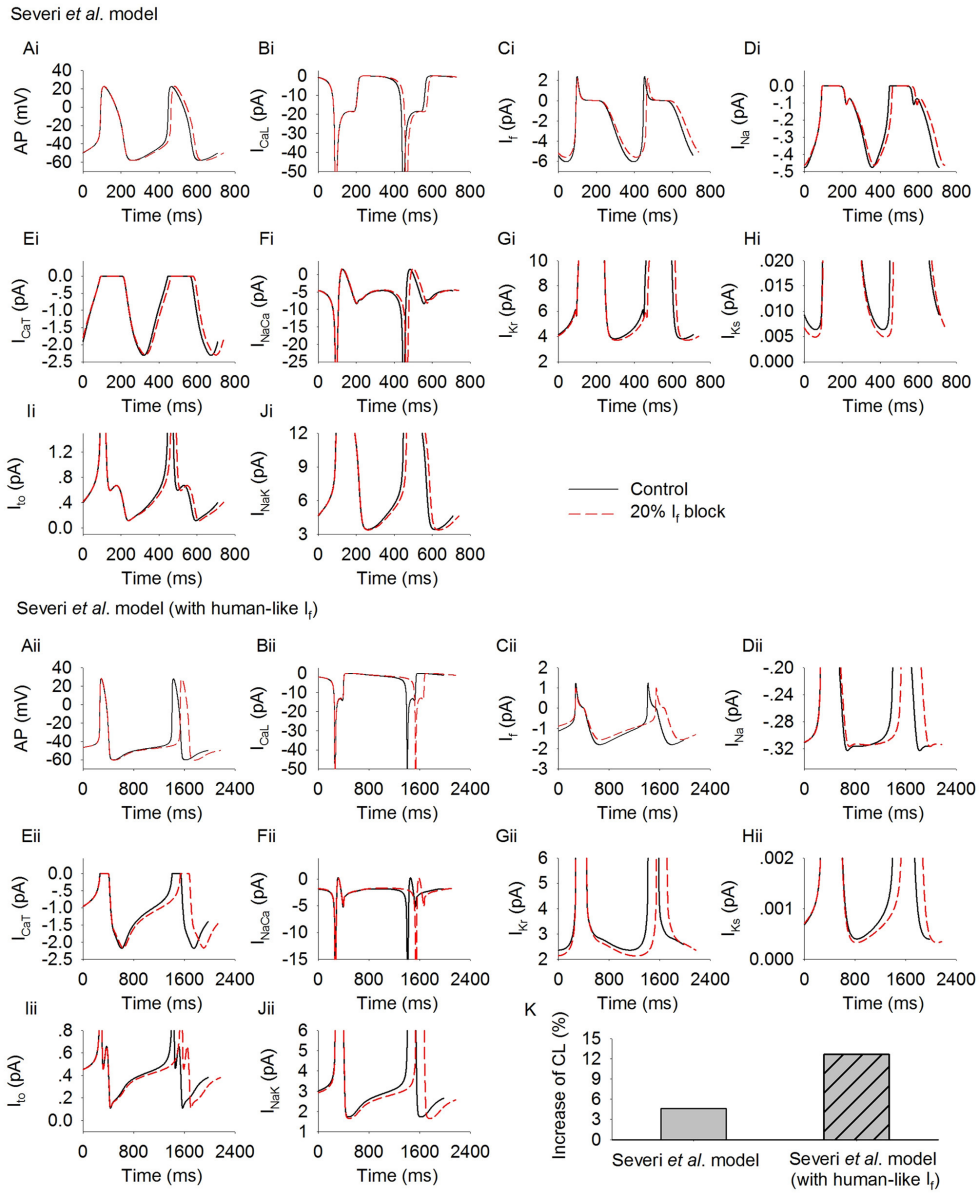
Simulated spontaneous action potentials and underlying currents by the Severi et al. model with rabbit *I*_f_ **(Ai–Fi)** and human *I*_f_ **(Aii–Fii)** formulations. Effects of blocking *I*_f_ by 20% on action potentials and underlying ionic currents (dotted lines) are also shown and superimposed with those in control condition (solid lines). **(Ai,Aii)**: action potentials; **(Bi,Bii)**: *I*_CaL_; **(Ci,Cii)**: *I*_f_; **(Di,Dii)**: *I*_Na_; **(Ei,Eii)**: *I*_CaT_; **(Fi,Fii)**: *I*_NaCa_; **(Gi,Gii)**: *I*_Kr_; **(Hi,Hii)**: *I*_Ks_; **(Ii,Iii)**: *I*_to_; **(K)** increase of pacing CL.

Figure 3 shows the simulated action potentials from the Severi et al. model with rabbit (Figures 3Ai–Fi) and human (Figures 3Aii–Fii) *I*_f_ formulations. In the figure, action potentials (Figures 3Ai,Aii) are shown together with membrane currents during the genesis of action potentials, including *I*_CaL_ (Figures 3Bi,Bii), *I*_f_ (Figures 3Ci,Cii), *I*_Na_ (Figures 3Di,Dii), *I*_CaT_ (Figures 3Ei,Eii), *I*_NaCa_ (Figures 3Fi,Fii), *I*_Kr_ (Figures 3Gi,Gii), *I*_Ks_ (Figures 3Hi,Hii), *I*_to_ (Figures 3Ii,Iii), and *I*_NaK_ (Figures 3Ji,Jii). By replacing the rabbit *I*_f_ formulation with the human *I*_f_ formulation, the pacemaking activity was slowed down, with a pacemaking CL that increased from 355 ms to 1,139 ms, which was associated with a slight increase in *I*_Na_ and *I*_CaT_ at the late period of the diastolic depolarization phase (DDP) (with no noticeable difference at the initial period of the DDP), and a slight decrease in *I*_Kr_, *I*_Ks_, *I*_to_, and *I*_NaK_ during the DDP. Such a small increase in the above-mentioned inward currents and a decrease in the outward currents, though contributory factors, are not the major determinants of the prolonged diastolic depolarization phase. The slowing down in the pacemaking activity of the human-like formulation model is mainly attributable to the smaller *I*_f_, *I*_CaL_, and *I*_NaCa_ during the diastolic depolarization phase.

In Figure 3, effects of reducing *I*_f_ by 20% on the pacemaking activity of the two models are also shown.

Reduction of *I*_f_ by 20% produced an increase of the CL in the rabbit-like *I*_f_ formulation model by about 4.6% (Figure 3K). This is in agreement with experimental data from isolated rabbit SAN cells, which showed that a low level of *I*_f_ block by ivabradine [<0.5 μM, a concentration not affecting SAN *I*_CaL_ which only slightly decreased by 18.12 ± 0.66% at 10 μM (Bois et al., 1996)] produced only a slight slowing down of the pacemaking rate, while >50% *I*_f_ blockade by 3 μM ivabradine (see Figure 1F) only reduced the spontaneous pacing rate by 11–17.7% (Bucchi et al., 2007; Yaniv et al., 2012).

However, in the human-like *I*_f_ formulation model, *I*_f_ reduction by 20% produced a more than 2-fold increase in the pacemaking CL by 12.4% (i.e., equivalent to about 11.1% reduction in the heart rate) as compared to the rabbit-like model (Figure 3K). The pacing rate reduction though was slightly less than the effect of intravenous administration of ivabradine by 0.2 mg·kg^−1^ (~23.5–34.1 nM of mean maximal ivabradine plasma levels) produced a reduction of heart rate by 18–20% (i.e., mean heart rate reductions of 12.9 and 14.1 beats min^−1^; Camm and Lau, 2003; Jiang et al., 2013), but close to experimental data of the pacing rate reduction when *I*_f_ was blocked by 3 μM ivabradine in rabbit SAN (Bucchi et al., 2007; Yaniv et al., 2012). This illustrates that the small human-like *I*_f_ has a greater effect on slowing down the pacing rate than the rabbit-like one when *I*_*f*_ is inhibited by ivabradine.

The results above suggested that the increased CL induced by 20% *I*_f_ reduction is proportional to the intrinsic cycle length of the model, i.e., the greater the intrinsic CL (e.g., the model with human-like *I*_f_ formulation) the greater the increase of the CL. This observation was model-independent as shown in Supplementary Figure 3, in which Fabbri et al. model of the human SAN was implemented by using rabbit-like and human-like *I*_f_ formulations. In the basal condition (Fabbri et al. model with human-like *I*_f_ formulation), the CL was 810 ms, which was increased by 44 ms with 20% *I*_f_ reduction (i.e., 5.5%). When the rabbit-like *I*_f_ formulation was used, the pacemaking rate was increased due to a larger *I*_f_, resulting a CL of 355 ms. With 20% *I*_f_ reduction, the CL was increased by 18 ms (i.e., 4.7%), which was smaller than that when the human-like *I*_f_ formulation was used.

As shown in Figures 3Ci,Cii, a 20% reduction in the channel conductance did not necessarily produce 20% reduction in *I*_f_ amplitude during the time course of action potential, due to the dependence of *I*_*f*_ on membrane voltage. To further investigate this, we computed the average *I*_f_ during the diastolic phase before and after 20% reduction in its channel conductance. Results are shown in Supplementary Figure 2. It was found that 20% reduction in the channel conductance produced a similar decrease in the avarage *I*_f_ in the rabbit-like (by 0.34 pA) and the human-like model (by 0.22 pA), but the relative change was greater in the latter model because of its smaller *I*_f_ in the control condition. Such difference in the relative change of *I*_f_ may also be one of the important reasons for the more pronounced prolongation of the diastolic phase in model with human *I*_f_ formulation. Note that in both models (rabbit-like and human-like models), the relative change of *I*_f_ was smaller than 20% though the channel conductance was reduced by 20%, due to the voltage-dependence of the channel's activation.

**Figure 4:**
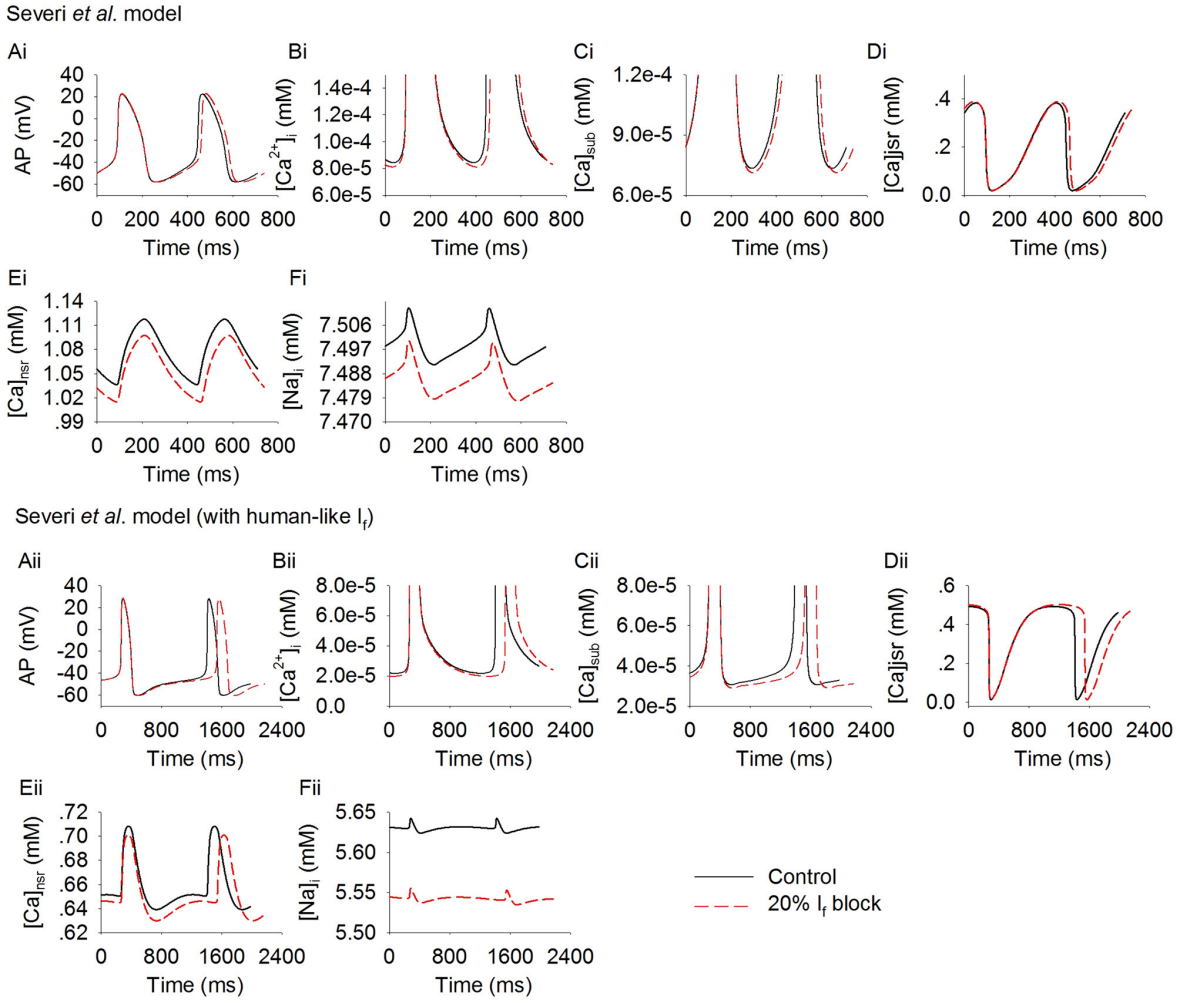
Effect of *I*_f_ block on the Ca^2+^ transient in rabbit-like **(Ai–Ei)** and human-like **(Aii–Eii)** SAN cell models. **(Ai,Aii)**: the action potentials; **(Bi,Bii)**: Ca^2+^ transient in the myoplasmic space; **(Ci,Cii)**: Ca^2+^ transient in the myoplasmic sub-space; **(Di,Dii)**: Ca^2+^ transients in the junctional SR; **(Ei,Eii)**: Ca^2+^ transient in the network SR space; **(Fi,Fii)**: intracellular Na^+^ concentration. Simulation results in control (solid line) are superimposed on those of *I*_f_ blocking (dotted lines).

Further simulations were carried out to analyze possible effects of the cross talk between the membrane clock (*I*_f_) and Ca^2+^ clock on modulating pacemaking action potentials in response to *I*_f_ reduction. Figure 4 shows results for the rabbit-like (Figures 4Ai–Ei) and human-like (Figures 4Aii–Eii) *I*_f_ formulation models. Blocking *I*_f_ by 20% decreased intracellular (cytoplasmic) Ca^2+^ transients ([Ca^2+^]_i_, Figures 4Bi,Bii), the intracellular subspace Ca^2+^ concentration ([Ca^2+^]_sub_, Figures 4Ci,Cii) and the Ca^2+^ content in the network SR ([Ca^2+^]_nsr_, Figures 4Ei,Eii). The diastolic level of [Ca^2+^]_sub_ was reduced by 2.9 and 5% in the rabbit-like and human-like *I*_f_ formulation model respectively, the decreased [Ca]_sub_ during the diastolic depolarization phase of the action potential (Figures 4Ci,Cii) leads to a decreased *I*_NaCa_ (Figures 3Fi,Fii) and *I*_CaL_ (Figures 3Bi,Bii), especially during the later phase of diastolic depolarization, which prolonged the later phase of the diastolic depolarization in the human-like *I*_f_ formulation model (Figure 4Aii). There was a negligible change in *I*_Na_ (Figures 3Di,Dii) and *I*_CaT_ (Figures 3Ei,Eii) during the diastolic phase. Taken together with the observation of changes in *I*_NaCa_ (Figure 3F), our simulation results imply that the slowing down of the spontaneous action potentials in response to a low level of *I*_f_ block (<20%) may be mainly attributable to *I*_f_ reduction (i.e., membrane clock), with some contribution from a crosstalk between the membrane clock and Ca^2+^ clock in the later diastolic depolarization phase.

**Figure 5:**
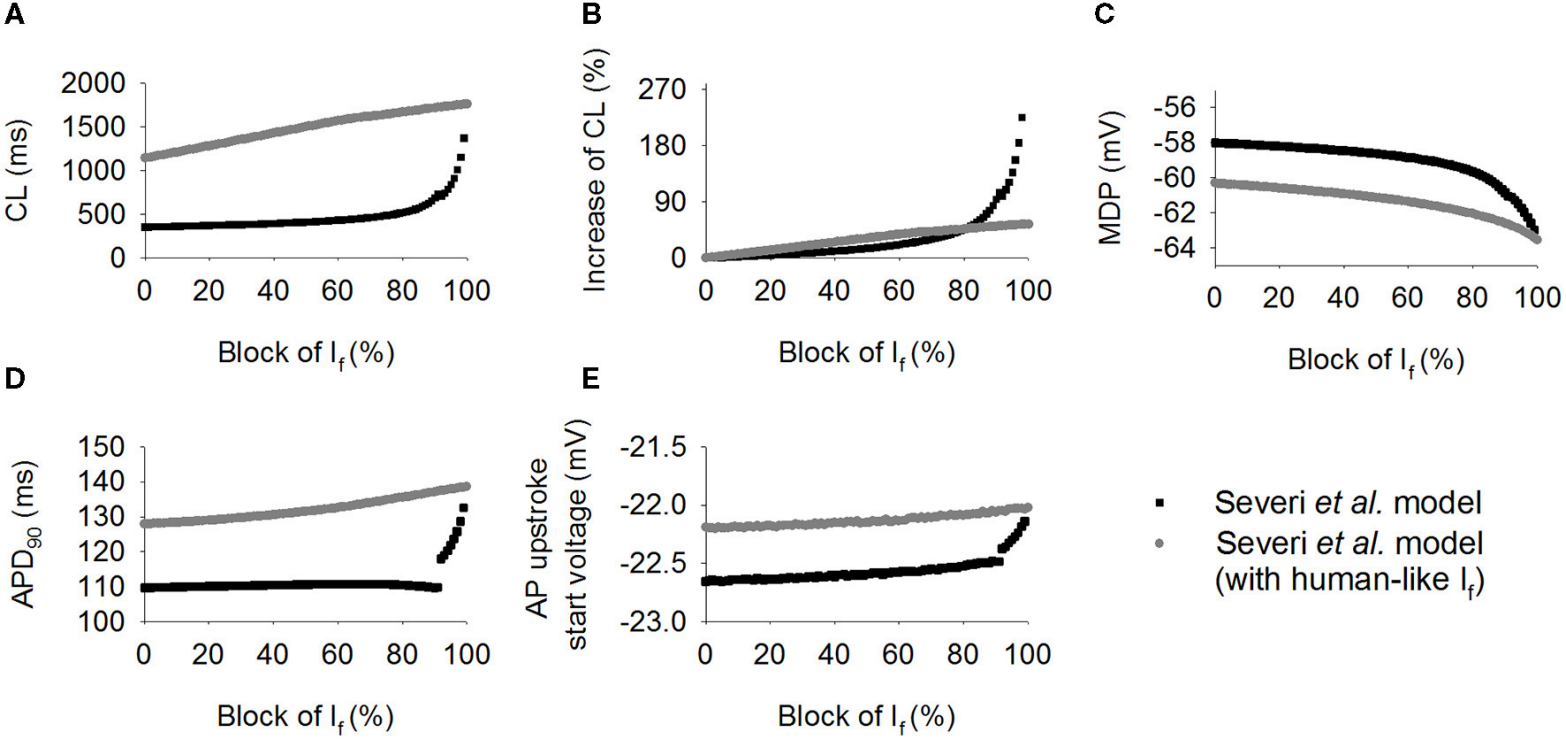
Simulated effect of *I*_f_ block on the CL increase. **(A,B)** Pacing cycle length and the relative percentage changes of CL when *I*_f_ blocked from 0 to 100% with 1% increment in the human-like (open circle) and rabbit-like (closed circle) SAN cell models; **(C,D)** the maximum diastolic potential (MDP) and action potential duration at 90% of repolarization (APD_90_); **(E)** change of the beginning of action potential upstroke voltage, which was calculated as the diastolic depolarization rate reached about 50% of its maximum value. *I*_f_ block produced greater effect in the human-like (gray circle) and rabbit-like (black square) SAN cell models.

Effects were also investigated of how a systematic change in *I*_f_ density affects spontaneous pacemaker activity. Results are shown in Figure 5, in which the computed CL (Figure 5A) and its increase (Figure 5B) with *I*_f_ being blocked from 0 to 100% with 1% increment for the rabbit-like (closed circles) and human-like *I*_f_ formulations (open circles). It was shown that over the range 0–80%, *I*_f_ block produced a greater CL increase with the human-like formulation than in the rabbit-like model. With a low level of *I*_f_ reduction, a linear relationship between the CL increase and *I*_f_ block was seen (Figure 5B). However, the relationship changed to an exponential one (Figure 5B) when a high level of *I*_f_ block was implemented. With about 66% *I*_f_ reduction, the CL was increased by about 26% (reduced pacemaking rate by 21%) in the rabbit-like model, which is consistent with experimental data (Thollon et al., 1994; Bucchi et al., 2007), but by about 42% in the human *I*_f_ formulation. A low level of *I*_f_ reduction (<20%) resulted in a negligible change in the MDP (<-0.3 mV in both models) in both rabbit-like and human-like *I*_f_ formulation models, and 100% *I*_f_ reduction hyperpolarised MDP by only 3.2 mV in the latter model (Figure 5C). Corresponding changes in APD_90_ (Figure 5D) and the voltage at the beginning of AP upstroke (Figure 5E) were also shown (also see Supplementary Table 1). While a high level of *I*_f_ reduction hyperpolarized the MDP, it had modest secondary effect on APD_90_ (mainly prolonging the late repolarization phase which may be due to the decreased *I*_Kr_ and *I*_NaK_ as shown in Supplementary Figures 4Gi,Ji, and there was no significant change in the fundamental morphology of the action potential as shown in Supplementary Figure 4Ai) and AP upstroke potential in the rabbit-like model.

Changes of the membrane currents and ion concentrations with a systematic change in *I*_f_ density in the two models were also investigated (Supplementary Figures 3, 4). During the diastolic depolarization phase of the action potentials, [Ca]_sub_ decreased as *I*_f_ density gradually decreased (Supplementary Figures 3Ci,Cii), further leading to a slower activation of the *I*_CaL_ (Supplementary Figures 4Bi,Bii) and a decrease in *I*_NaCa_ (Supplementary Figures 4Fi,Fii) during this phase. Reduction *of I*_NaCa_ decreased [Na]_i_ (Supplementary Figures 3Fi,Fii), leading to a reduction in *I*_NaK_ (Supplementary Figures 4Ji,Jii) which further exacerbated the reduction of [Na]_i_. The outward currents (*I*_Kr_, *I*_Ks_, *I*_to_, *I*_NaK_) also showed a gradual decrease with a decreased *I*_f_ density during the diastolic depolarization phase (Supplementary Figures 4Gi–Ji,Gii–Jii).

The simulated action potentials from the two models showed some differences when *I*_f_ was fully blocked (Figure 5A), with the rabbit-like model failing to generate spontaneous action potentials. Such differences may be attributable to the use of different sets of initial values recorded from their steady state variables as there was no other changes in model equations or parameters, except for the use of rabbit-like *I*_f_ or human-like *I*_f_ formulations. In order to determine potential factors contributing to such differences or contributing to the pacemaking action potentials in the two models, further analyses on membrane currents and the intracellular Ca^2+^ transients between control and 99% reduction of *I*_f_ were conducted. Results are shown in Supplementary Figures 3, 4. It was shown that in both models, in addition to *I*_f_, I_CaT_, I_NaCa_, and I_Na_ contributed to the diastolic depolarization. With a high level of *I*_f_ reduction (99%), there was a significant decrease in [Ca]_sub_ during the diastolic depolarization phase (Supplementary Figure 3Ci), causing a significant reduction in *I*_NaCa_ (Supplementary Figure 4Fi). Consequently, the spontaneous membrane depolarization was not able to reach the *I*_CaL_ activation potential, terminating the action potentials in the rabbit-like model.

In the human-like model, a reduction of [Ca]_sub_ was also observed with a high level of *I*_f_ reduction, resulting in a decreased *I*_NaCa_. However *I*_NaCa_ was sufficient to maintain the spontaneous depolarization to generate a full action potential.

The focus of this study was on the action of a modest extent (i.e., <20%) of *I*_f_ bock, mimicking the clinical use of ivabradine, rather than on the action of a large percentage of *I*_f_ block. With <50% *I*_f_ block, the increase in CL was about <30%, which is reasonably close to the experimental data observed in rabbit SAN cells when *I*_f_ is blocked by use of Cs^+^ (Nikmaram et al., 1997), validating the physiological relevance of the results obtained.

**Figure 6:**
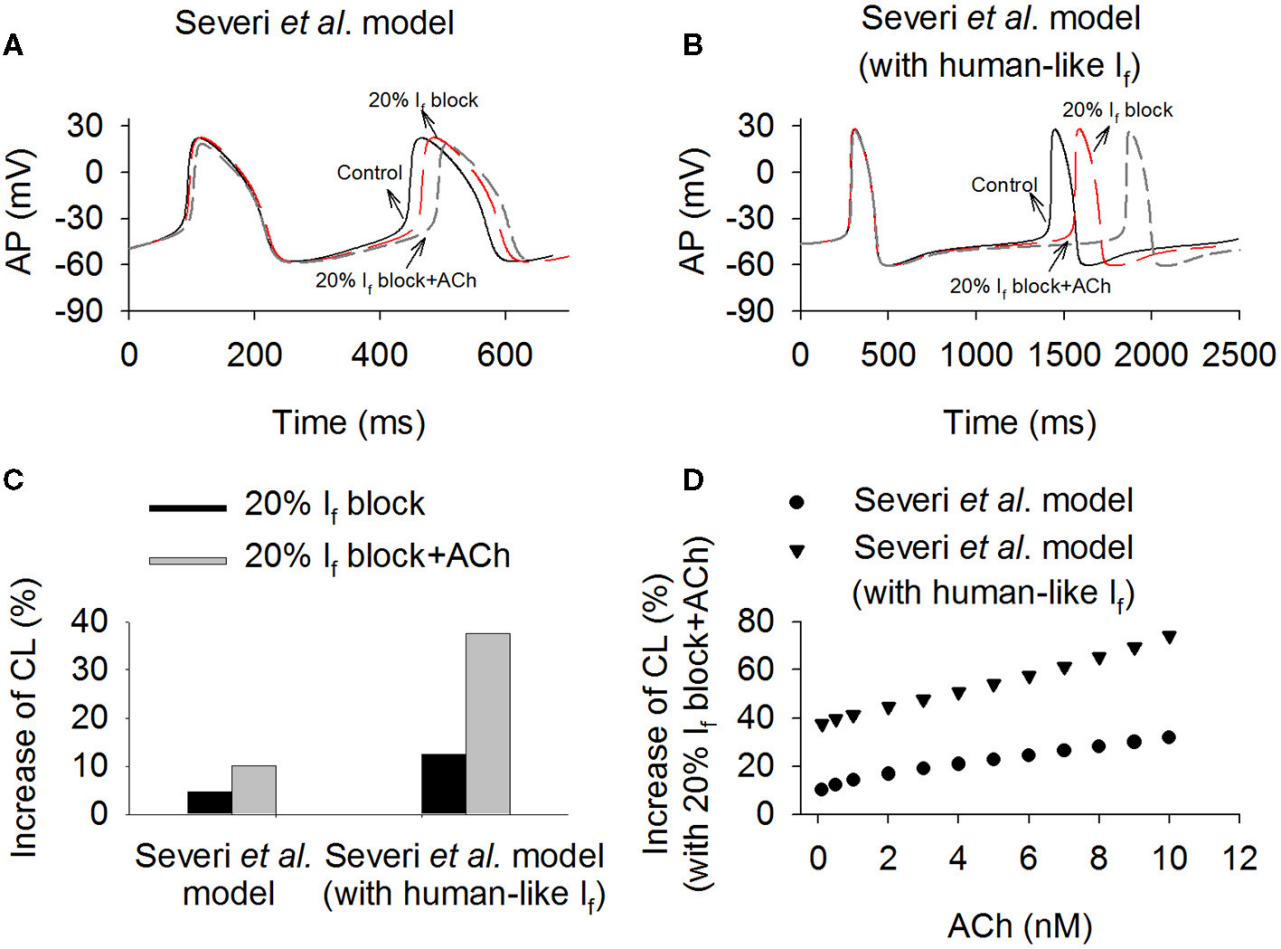
Effects of combined action of ACh and 20% *I*_f_ reduction on sponteneous action potentials in the Severi et al. model with the rabbit-like and human-like *I*_f_ formulations. **(A,B)** Time courses of action potentials in control, *I*_f_ reduction and combined effect of *I*_f_ reduction and ACh (0.1 nM). **(C)** The increase of pacemaking CL in the conditions of 20% *I*_f_ block alone and combined action with ACh (0.1 nM). **(D)** Combined effect of variant concentrations of ACh (from 0.1 to 10 nM) and 20% *I*_f_ block on pacemaking CL in the rabbit-like and human-like *I*_f_ formulations models.

Further simulations were conducted to investigate the combined action of *I*_f_ reduction by ivabradine and actions of ACh (Boyett et al., 1995), mimicking the autonomic regulation of cardiac pacemaking *in vivo*. In simulations, acetylcholine-dependent inhibition of *I*_f_ and *I*_CaL_, and activation of K^+^ current (*I*_K,ACh_) (Voigt et al., 2014) were considered. Results are shown in Figure 6, in which computed time courses of APs in control (with both rabbit-like and human-like *I*_f_ formulations), *I*_f_ reduction alone (by 20%) and *I*_f_ reduction together with actions of 0.1 nM ACh were compared (Figures 6A,B). It was shown that ACh augmented the effect of *I*_f_ reduction on the increase of CL in the model with both the rabbit-like and human-like *I*_f_ formulations. With the action of 0.1 nM ACh, 20% of *I*_f_ block increased the CL by 37.5% (about 27.3% reduction the pacing rate) (Figure 6C) in the model with human-like *I*_f_ formulation, which is remarkably greater than that of 10.1% (about 9.1% reduction in the pacing rat) in the model with the rabbit-like *I*_f_ formulation. This observation held true when different ACh concentrations were considered (Figure 6D). These results illustrate that the combined action of *I*_KACh_ and *I*_f_ reduction further slowed down the pacemaking AP due to a reduced total depolarization current during diastolic depolarization phase, resulting in a greater CL prolongation. It suggested that the clinical observed effect of low ivabradine on reducing pacing rate may partially result from the action of ACh due to active vagal tone *in vivo*. Similar observations were also seen in the Fabbri et al. model as shown in Supplementary Figure 4, in which ACh augmented more the effect of *I*_f_ reduction on pacemaking rate in the model with human-like *I*_f_ formulation than that with rabbit-like *I*_f_ formulation. Results from the Fabbri et al. model were similar, as shown in Supplementary Figure 5.

### Theoretical Analysis Validation

The simulation results presented above showed that *I*_f_ block produced a greater impact on slowing down the pacemaking rate with the human-like *I*_f_ formulation than that in the rabbit-like cell model in both the Severi et al. and Fabbri et al. model (see Supplementary Material). This is paradoxical as the *I*_f_ density over the pacemaker range with the human-like formulation is much smaller than that in the rabbit-like model, and one would expect a smaller *I*_f_ contribution to the spontaneous action potentials (therefore a smaller CL increase with *I*_f_ block). However, such a paradoxical effect of *I*_f_ reduction on the increase of CL as observed in the two models matched the theoretical prediction shown in the Method section (Equation 2), which showed an inverse relationship between an increased CL and the amplitude of total ionic membrane currents during the diastolic depolarization phase. With the theoretical prediction, a greater CL increase in the human-like *I*_f_ formulation SAN cell model can be attributed to a smaller *I*_total_ during the diastolic depolarization phase.

**Figure 7:**
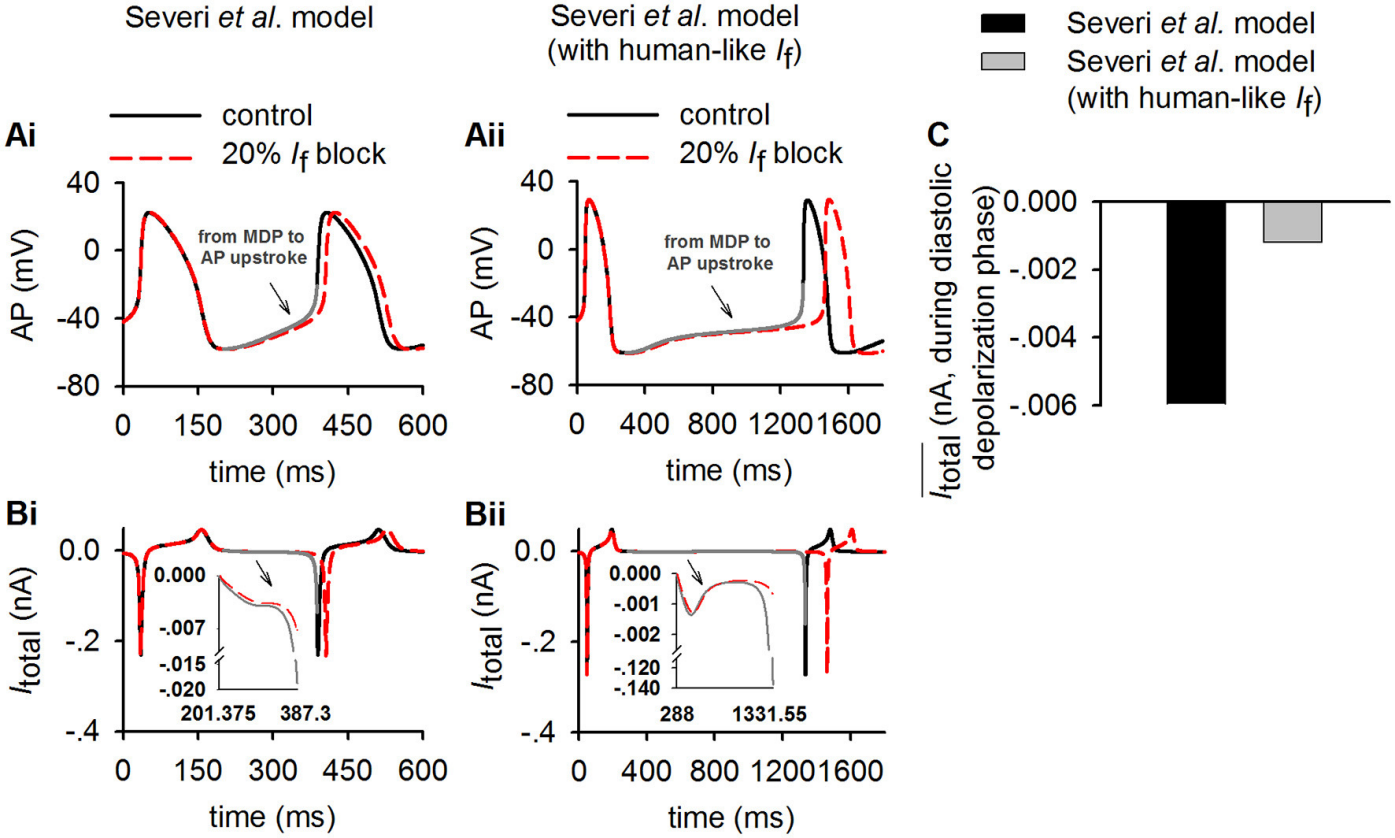
Simulation of the inverse relationship of *I*_total_ and the increasing of CL in control (solid lines) and 20% *I*_f_ reduction (dotted lines) to validate theoretical analysis result (Equation 2) in method section (*p* = 0.2, modeling 20% *I*_f_ reduction). **(Ai,Aii)** Time course of action potentials for rabbit-like and human-like SAN cell models respectively (gray lines represents diastolic depolarization voltage changes from the MDP to the voltage at the beginning of AP upstroke in control condition); **(Bi,Bii)** time courses of *I*_total_ (gray lines represents diastolic depolarization voltage changes from the MDP to the voltage at the beginning of AP upstroke in control condition). **(C)** Averaged value of *I*_total_ amplitude during the diastolic depolarization phase for the rabbit-like (black) and human-like (gray) SAN cell models. In each case, the integral interval was set to the time interval between the MDP and beginning of the upstroke membrane potential (V_up_).

To test the theoretical prediction, further analyses were conducted to compute the averaged *I*_total_ amplitude during the DDP. Results from the Severi et al. model are shown in Figure 7 for control and 20% *I*_f_ reduction for action potentials (Figures 7Ai,Aii), the time course of *I*_total_ (Figures 7Bi,Bii) and the averaged amplitude of *I*_total_ (Figure 7C) during the DDP. It was found that during the time course of diastolic depolarization, the averaged *I*_total_ amplitude in the cell model with the human *I*_f_ formulations was much smaller (<30%) than that in the rabbit-like model, which produced a slower pacemaking rate (i.e., longer CL) and greater CL increase in response to 20% *I*_f_ reduction, matching the theoretical prediction. The changes in currents and Ca^2+^ ion concentration associated with the change in *I*_*total*_ (Figures 7Bi,Bii) are shown in Figures 3, 4. A similar observation was also seen in the Fabbri et al. model as shown in Supplementary Figure 6C.

**Figure 8:**
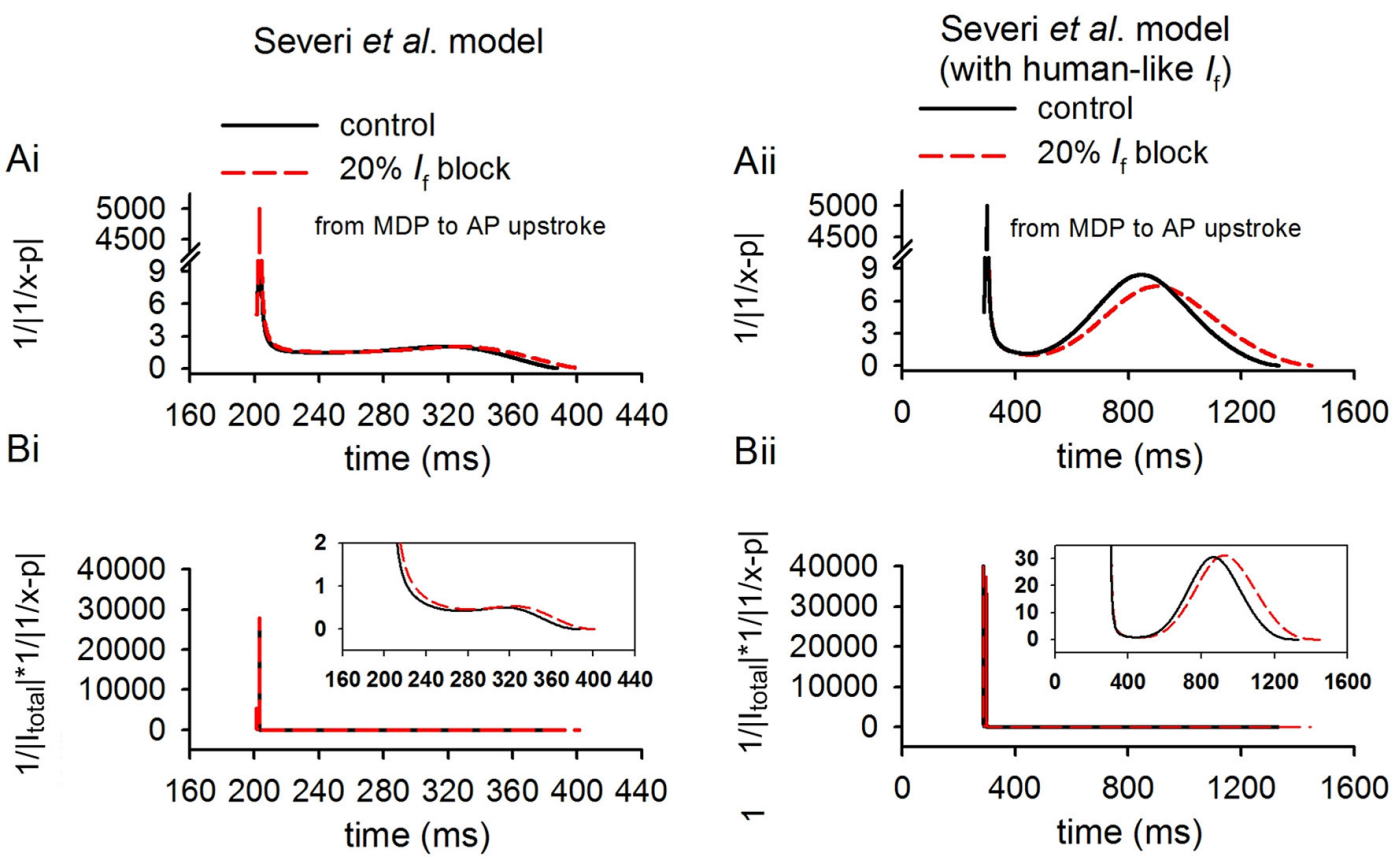
Computed values of $\frac{1}{\left|1/x-p\right|}$ and $\frac{1}{\left|I_{total}\right|}\cdot \frac{1}{\left|1/x-p\right|}$ to validate theoretical analysis result (Equation 2) in method section (*p* = 0.2, modeling 20% *I*_f_ reduction). The highlighted lines shown in this figure are during the diastolic depolarization phase of the action potentials of control (solid lines) and 20% *I*_f_ reduction (dotted lines) cases for the rabbit-like **(Ai,Bi)** and human-like **(Aii,Bii)** SAN cell models.

In theoretical analysis it was shown that the relative increase of CL was also influenced by a factor of $\frac{1}{\left|1/x-p\right|}$, which was related to the ratio of *I*_f_ to *I*_total_ (*x*) and *I*_f_ block potency (*p*). In simulations, we further computed the values of $\frac{1}{\left|1/x-p\right|}$ and $\frac{1}{\left|I_{total}\right|}\cdot \frac{1}{\left|1/x-p\right|}$ for control and *I*_f_ reduction cases. Results from the Severi et al. model are shown in Figure 8, in which the time courses of $\frac{1}{\left|1/x-0.2\right|}$ (Figures 8Ai,Aii) and $\frac{1}{\left|I_{total}\right|}\cdot \frac{1}{\left|1/x-0.2\right|}$ (Figures 8Bi,Bii) were plotted for the control (black) and 20% *I*_f_ reduction (*p* = 0.2) for the rabbit-like (left panels) and human-like (the right panels) models. The computed $\frac{1}{\left|1/x-0.2\right|}$ (acting as a piecewise function) was set to $\frac{1}{\text{1}\times \text{1}{\text{0}}^{\text{-}3}}$ when |1/*x* − 0.2| was smaller than 1 × 10^-3^ to avoid the value close to positive infinity. It was shown that in both models with a small *I*_f_ block the difference in the computed value of $\frac{1}{\left|1/x-p\right|}$between control (solid line) and 20% *I*_f_ reduction (dotted line) was very small during diastolic depolarization phase, and also small when the value was normalized against *I*_total_ ($\frac{1}{\left|I_{total}\right|}\cdot \frac{1}{\left|1/x-0.2\right|}$). This provides support for the notion that the relative increase of CL was mainly determined by the amplitude of *I*_total_ during the diastolic depolarisation phase in response to *I*_f_ block.

Note that during the last period of diastolic depolarisation phase (i.e., during time period of 310–387 ms as shown in the figure for the rabbit-like model and 890–1,331 ms for the human-like model), the difference in the computed values of $\frac{1}{\left|1/x-0.2\right|}$ and $\frac{1}{\left|I_{total}\right|}\cdot \frac{1}{\left|1/x-0.2\right|}$ became more noticeable. This may be attributable to the different timings by which the upstroke of pacemaking actions potentials occurred between control and *I*_f_ reduction conditions. Note that the value of $\frac{1}{\left|1/x-0.2\right|}$was also greater in the human-like model than that in the rabbit, which amplified the contribution of $\frac{1}{\left|I_{total}\right|}$toward a relative increase of CL. Results from the Fabbri et al. model were similar, as shown in Supplementary Figure 7.

**Figure 9:**
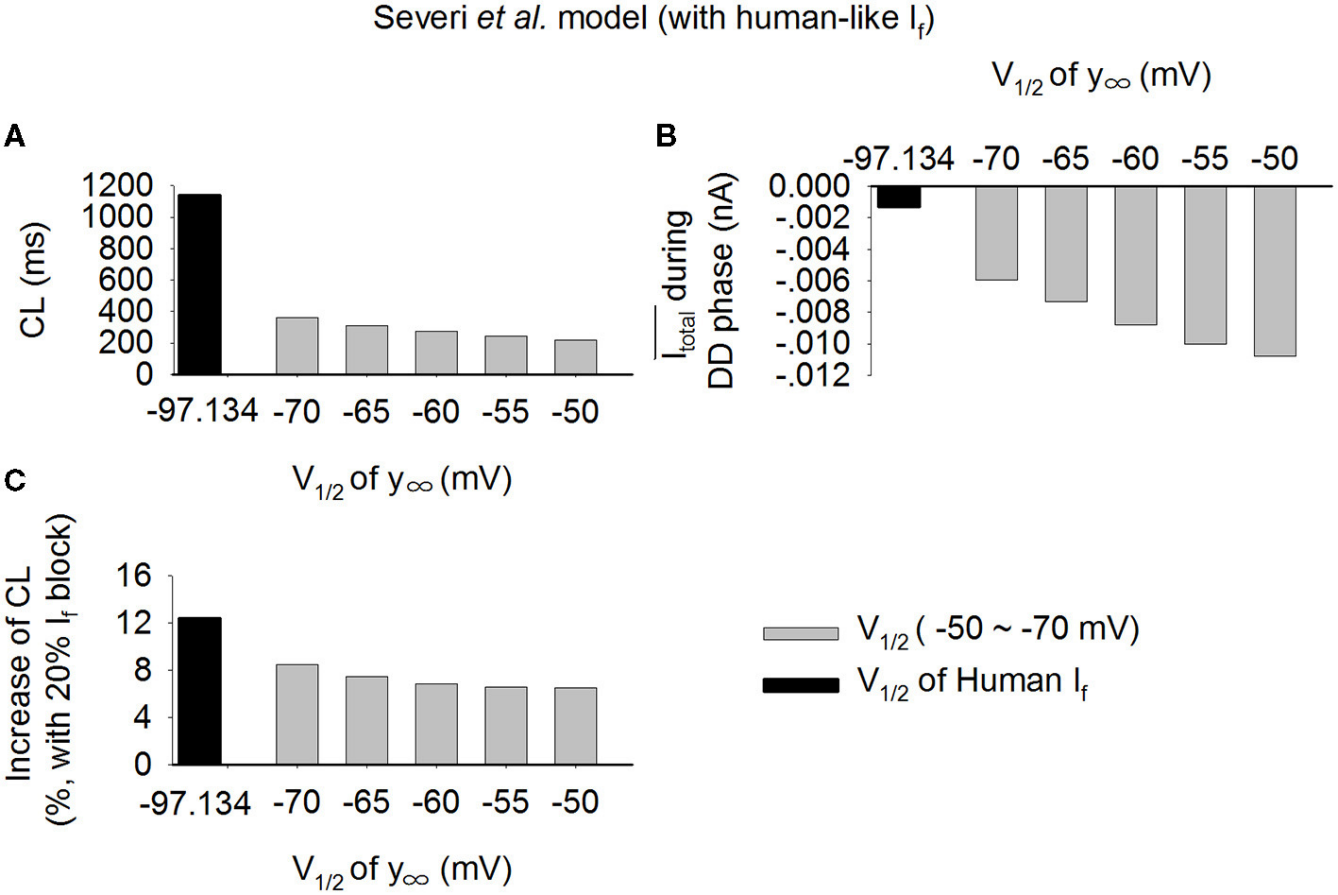
**(A–C)** Effect of varied half-activation voltage (*V*_1/2_) of the steady state activation variable (y_∞_) on pacemaking CL in the Serveri et al. model with human-like *I*_f_ formulation. *V*_1/2_ changed from −50 to −70 mV, covering the experimental data range for rabbit (−50 mV) and human (−97.1 mV) SAN cells.

A marked difference in the *V*_1/2_ of the steady-state activation relationship (y_∞_) of *I*_f_ between the rabbit and the human SAN cells has been observed (Difrancesco et al., 1989; Altomare et al., 2003; Barbuti et al., 2007; Verkerk et al., 2007b). In this study, we used *V*_1/2_ of −52.5 and −97.1 mV for the rabbit-like and the human-like *I*_f_ formulation respectively. In order to systematically determine a possible role of varying *V*_1/2_ in modulating *I*_f_ amplitude, and thus the *I*_total_ and the effect of *I*_f_ reduction on the increase of CL, we changed *V*_1/2_ of y_∞_ in the human-like *I*_f_ formulation in a border range from −50 to −70 mV. Results from the Serveri et al. model are shown in Figure 9 for the pacemaking CL (Figure 9A), the averaged *I*_total_ during diastolic depolarization phase (Figure 8B), and increase of CL (Figure 9C) with 20% *I*_f_ block. Shifting the *V*_1/2_ from −50 mV (about rabbit V_1/2_) to −70 mV (about human V_1/2_), the *I*_total_ was decreased (Figure 9B), which were correlated with an increased CL (Figure 9A) as well as an increased effect of *I*_f_ reduction on CL (Figure 9C). These results supported our theoretical analysis on that a smaller *I*_f_ in the model resulted in a smaller *I*_total_ amplitude, resulting in a slower pacemaking rate; and as such the same reduction in *I*_f_ resulted in a more significant change of CL in the cell model with a smaller *I*_total_. Similar results were also observed using the Fabbri et al. model as shown in Supplementary Figure 8.

## Discussion

This study was conducted to determine the mechanism by which a low level of *I*_f_ block by clinical drug (ivabradine) concentration is able to reduce the heart rate of patients by about 18–20%. In a previous study, Verkerk and Wilders found that though *I*_f_ has a small magnitude in the human SA node cells, it has an equally important role as in the rabbit (Verkerk and Wilders, 2010). In another study, Maltsev and Lakatta argued that *I*_f_ provides a relatively modest contribution to spontaneous beating rate regulation of human and rabbit sinoatrial node cells, and its major role in human SAN cells is to prevent excessive hyperpolarization during AP repolarization, thus representing an anti-bradycardic mechanism, rather than a primary rate regulatory mechanism (Maltsev and Lakatta, 2010). Though these previous studies addressed how a smaller *I*_*f*_ can produce the same effect in humans with respect to rabbit (Verkerk and Wilders, 2010; Fabbri et al., 2017) on complete *I*_*f*_ block, however, the question on how <20% *I*_*f*_ block produces a marked heart rate reduction in humans in *vivo* had remained unclear. This is due to the nonlinear relationship between the extent of *I*_*f*_ block and heart rate reduction (see Figure 5); effects of partial *I*_*f*_ block cannot be predicted with certainty from the complete block data already in the literature.

The principal contributions of the work are insights into how a small extent of *I*_*f*_ block (<20%; as may occur during clinical use of ivabradine) produces a marked heart rate reduction in human SAN, greater than that predicted by single cell experiments in the rabbit though the latter has a greater *I*_*f*_ density. Our principal findings are: (i) blocking *I*_f_ by 20% resulted in only about a 4.6 % increase in the CL in the rabbit-like SAN cell model, but about 12.4% in the cell model with human *I*_f_ formulation. This finding suggests that *I*_f_ block has a greater effect with the human-like SAN cell *I*_f_ formulation than with rabbit-like *I*_f_ formulation, despite the fact that the former has a smaller *I*_f_ current density over diastolic potentials, based on which one might expect a smaller contribution of *I*_f_ to pacemaking; (ii) there is a cross-talk between the membrane clock and Ca^2+^ clock with 20% *I*_f_ block in the later phase of the diastolic depolarization of the action potential; (iii) a theoretical analysis matching the simulation data has been produced, providing a numerical formalism explaining the relationship between *I*_*f*_ block effects and its contribution to total current during diastolic phase. In this study, both numerical simulations and theoretical analysis here have attributed the paradoxical effect of *I*_f_ reduction (i.e., a greater effect in SAN cells with smaller *I*_f_ current density and therefore a slower heart rate) to an inverse relationship between the relative increase of CL and the amplitude of the total current during the diastolic depolarization phase in response to *I*_f_ block; and (iv) significantly, vagal tone activity *via* ACh augments the effects of ivabradine on heart rate reduction, providing a possible mechanism(s) by which the clinical concentrations can have larger effects *in vivo* than those predicted by single cell experiments *in vitro*. It has been shown that combined action of ACh (0.1 nM) and 20% *I*_f_ reduction markedly increase the pacemaking CL by 37.5%, close to the clinical effect of ivabradine when human-like *I*_f_ formulation was used, which is significantly greater than that of 10.1% when the rabbit-like *I*_f_ formulation was used. These findings were also observed in the Fabbri et al. model.

The results of the present study demonstrate and explain why a smaller human *I*_f_ has a greater effect on prolonging the diastolic depolarization phase when it is partially blocked, using both mathematical theoretical analysis and computer simulation, which is clearly distinct from the previous studies (Verkerk and Wilders, 2010; Fabbri et al., 2017). Collectively, they add mechanistic insight into the understanding of how a low dose of clinical use of ivabradine (<137 nM) can effectively slow down the human heart rate by about 18–20% (Camm and Lau, 2003; Joannides et al., 2006; Doesch et al., 2007; Jiang et al., 2013), which contrasts with a negligible effect predicted by experimental studies in the rabbit (<4% at 10 min after administration of ivabradine; Thollon et al., 2007).

### Role of *I*_f_ in Generating Cardiac Pacemaking Activity

*I*_f_ channels encoded by HCN genes are richly expressed in cardiac conduction systems including the primary pacemaker, the SAN (Altomare et al., 2003; Ravagli et al., 2016). Previous studies from animal models have suggested an important role of *I*_f_ in the SAN (Difrancesco and Noble, 2012; Baruscotti et al., 2016; Kozasa et al., 2018). It has been shown that a complete block of *I*_f_ by Cs^+^ (2 mM) slowed down the pacemaking rate by 30% in the rabbit SAN cells (Denyer and Brown, 1990), a 17.6% reduction of pacemaking rate also seen when blocking of *I*_f_ with 0.5 mmol/L Cs^+^ (Liu et al., 2008). In transgenic mice, knocking down HCN4 produced bradycardiac effects as well as atrioventricular node conduction block (Herrmann et al., 2007; Hoesl et al., 2008; Baruscotti et al., 2011). All of this evidence demonstrates that *I*_f_, together with the more recently identified Ca^2+^ clock [arising from the coupling between the intracellular Ca^2+^ cycling and electrogenesis of membrane currents (e.g., *I*_NaCa_) (Maltsev and Lakatta, 2008; Lakatta and Difrancesco, 2009)], provide a major driving force for generating the spontaneous depolarization during the diastolic phase that leads to intrinsic pacemaker activity.

*I*_f_ is also present in human SAN cells (Verkerk et al., 2009b; Li et al., 2015) and contributes to pacemaking. It has been shown that loss-of-function of HCN channel mutations is associated with sick sinus syndrome, which manifests with symptoms of bradycardia and conduction block (Schweizer et al., 2014; Verkerk and Wilders, 2014, 2015). However, the functional role of *I*_f_ in generating pacemaking action potentials of human SAN is less well-characterized as compared to that from small mammals. Limited data have shown that *I*_f_ in the human SAN cells has a smaller current density, more negative depolarised membrane potential of half maximal activation and slower activation rate as compared to the rabbit (Verkerk et al., 2007a,b, 2009b). All of these suggested a smaller *I*_f_ current during the diastolic depolarization phase, which may result in a slower heart rate. Indeed this is the case as shown in the present simulation study. In the Severi et al. model (Severi et al., 2012) with the rabbit-like *I*_f_ formulation, the measured pacemaking cycle length was 354.8 ms. However, when the rabbit-like *I*_f_ formulation was replaced by the human one, the pacemaking rate was slowed down and the pacemaking cycle length increased to 1,139.4 ms, greater than the intrinsic pacemaking cycle length of native human SAN cells [about 828 ± 21 ms (Verkerk et al., 2007b)]. Note that in the model, such a significant increase of the pacemaking CL from the one of rabbit SAN cells to the one close to human SAN cells was mainly attributable to a small *I*_f_, as no change or negligible secondary changes of other ion channels were implemented. This suggests that *I*_f_ exerts a strong influence on pacemaker rhythm. Note that there is a marked difference in the pacemaking CL between the human-like model (1,139.4 ms) and native human SAN cells [about 828 ± 21 ms (Verkerk et al., 2007b)], which may be due to possible species differences in the properties of other membrane currents (Fabbri et al., 2017) and Ca^2+^ clock (Tsutsui et al., 2018). With the increase of *I*_f_ blocking, the pacemaking CL increased non-linearly in the model with either rabbit-like or human-like *I*_f_ formulations.

In simulations, the action of 100% *I*_f_ block abolished the pacemaking in the model with rabbit-like *I*_f_ formulations. This may over emphasize the role of *I*_f_ in the rabbit SAN pacemaking and is non-physiological. However, the focus of this study is on the action of small *I*_f_ block (i.e., <20%), mimicking the use of effect of ivabradine in practice, rather than on the action of large percentage of *I*_f_ block. With <50% *I*_f_ block, the increase in CL is about <30%, which is reasonably close to the experimental data observed in rabbit SAN cells when *I*_f_ is blocked by use of Cs^+^ (Nikmaram et al., 1997).

### Mechanism for the Action of Low Dose Ivabradine on Human SAN

Our theoretical and numerical simulation results have shown that a low level of *I*_f_ block, mimicking the clinical concentrations of ivabradine, produced a more marked effect in reducing the heart rate of the human-like *I*_f_ formulation SAN cell model than the rabbit-like *I*_f_ formulation SAN cell model. When the action of ACh (0.1 nM) was considered, there was a further reduction of sponteneous pacing rate (reduced upto by 27.3%), which is close to the effect of ivabradine at clinical concentration. Results from the Fabbri et al. model were similar, showing that these observations are model-independent. All these results suggest that a combined action of *I*_f_ reduction by ivabradine at clinical concentrations and ACh are attributable to the heart rate reduction as seen clinically (Camm and Lau, 2003; Doesch et al., 2007).

It is possible that ivabradine regulates the heart rate by a cross-talk between the membrane clock and Ca^2+^ clock *via* the electrogenic Na^+^-Ca^2+^ exchangers (Yaniv et al., 2013). While the direct action of ivabradine on the intracellular Ca^2+^ handling is unclear, our simulation results showed that a 20% *I*_f_ reduction produced secondary modulations of other ionic currents (e.g., *I*_NaCa_) and the intracellular Ca^2+^ handling, suggesting there is a cross-talk between the membrane clock and Ca^2+^ clock in the late phase of the diastolic depolarization of the action potential (Figures 4Bii–Eii). All these illustrated that ivabradine affected the heart rate at clinical concentrations mainly through regulating membrane clock and Ca^2+^ clock, and combining with the action of ACh.

### Limitations of the Study

Possible limitations of the Severi et al. model of the rabbit SAN cells have been well-discussed and documented (Severi et al., 2012). For example, as highlighted by Verkerk and Wilders (2014), the reversal potential of *I*_f_ in the Severi et al. model (−4.39 mV) was more positive than that experimentally reported (about −30 mV) (Difrancesco et al., 1986; Van Ginneken and Giles, 1991; Verkerk et al., 2009a), which is a limitation of the Severi et al. model, and therefore also of studies employing this model. However, we did not modify the *I*_f_ equation of the Severi et al. model for the following reasons: (1) the simulated I-V relationship data shown in (Figure 1C) lie within the range of experimental data, and *I*_f_ is very small when the voltage is more positive than −40 mV; (2) when the voltage is more positive than −30 mV, the activation of *I*_CaL_ is dominant, which contributes mainly to the upstroke phase (non-diastolic depolarization phase) of the action potential. Therefore, even if the reversal potential of *I*_f_ is changed from −30 mV to −4.39 mV, it has negligible effect on the diastolic depolarization of spontaneous action potentials. Moreover, in simulations in which the rabbit-like *I*_f_ was replaced by the human-like formulations in the Severi et al. model, the reversal potential of *I*_f_ used was −27.5 mV, close to the experimentally determined value of −22.1 ± 2.4 mV (Verkerk et al., 2007b).

Another potential limitation of the present study relates to modification of the Severi et al. model to incorporate the human *I*_f_ formulation developed by Fabbri et al. based on experimental data from human SAN cells. Due to lack of experimental data from human SAN cells, equations and parameters for other ion channels and transporters in the Severi et al. model were not updated. Note that experimental data on the Ca^2+^ clock of the human SAN became available recently (Tsutsui et al., 2018), which are not incorporated in the model yet. This may explain why the computed CL of the human-like SAN cell is greater than that of natural human SAN cells, and consequently the smaller effect of *I*_f_ blocking on the reduction of the heart rate the clinical data. In addition, the action of low dose ivabradine was simulated by considering its action on blocking *I*_f_ only, and did not incorporate its possible actions on *I*_Kr_ as seen in some experimental studies on ventricular cells/tissue at low dose (Melgari et al., 2015) or high doses (Lees-Miller et al., 2015). However, the present study deliberately focused on the difference in direct effects of ivabradine on *I*_f_ in the SAN cells between species (*I*_f_ in the rabbit SAN and in the human-like SAN). Whilst it is necessary to point out these potential limitations, nevertheless, the simulation data strongly supported the mechanism demonstrated by the theoretical analysis in showing the inverse correlation between *I*_total_ during the diastolic depolarization phase and the relative increase of the CL. Therefore, these limitations do not alter our major conclusion on the role of *I*_f_ block in modulating cardiac pacemaking activities in the human SAN by low concentrations of ivabradine.

## Conclusion

An inverse correlation between the relative increase of CL and the amplitude of the total ion channel current during the diastolic depolarization phase has been observed. Both theoretical analysis and simulations have shown that a low level of *I*_f_ block (<20%) can produce a more marked reduction of in the pacemaking rate of the human-like SAN cell model than the rabbit-like one due to its smaller *I*_total_ during the diastolic depolarization phase. This was particularly the case when ACh actions were considered, which amplified the pacemaking cycle length prolongation. This study thus provides a mechanistic explanation into how a low level of *I*_f_ block by the clinical concentrations of ivabradine can effectively reduce the heart rate in humans but produce a small or negligible effect in the rabbit.

## Data Availability Statement

The original contributions presented in the study are included in the article/Supplementary Material, further inquiries can be directed to the corresponding author/s.

## Code Availability Statement

The code for the models employed is available on request to Henggui Zhang at henggui.zhang@manchester.ac.uk.

## Author Contributions

HZ and JH conceived the study. XB conducted simulation and data analysis. HZ and MB contributed to theoretical analysis. XB, KW, MB, JH, and HZ wrote the paper. All authors contributed to the article and approved the submitted version.

## Conflict of Interest

The authors declare that the research was conducted in the absence of any commercial or financial relationships that could be construed as a potential conflict of interest.

## Publisher's Note

All claims expressed in this article are solely those of the authors and do not necessarily represent those of their affiliated organizations, or those of the publisher, the editors and the reviewers. Any product that may be evaluated in this article, or claim that may be made by its manufacturer, is not guaranteed or endorsed by the publisher.
