# Supplementary material for: The Functional Role of Hyperpolarization Activated Current (If) on Cardiac Pacemaking in Human vs. in the Rabbit Sinoatrial Node: A Simulation and Theoretical Study
Source: Front Physiol. 2021 Aug 19;12:582037. doi: 10.3389/fphys.2021.582037 (PMC8417414; doi:10.3389/fphys.2021.582037)
Supplement: Supplementary file 2 [file Image_2.pdf]

Supplementary Material

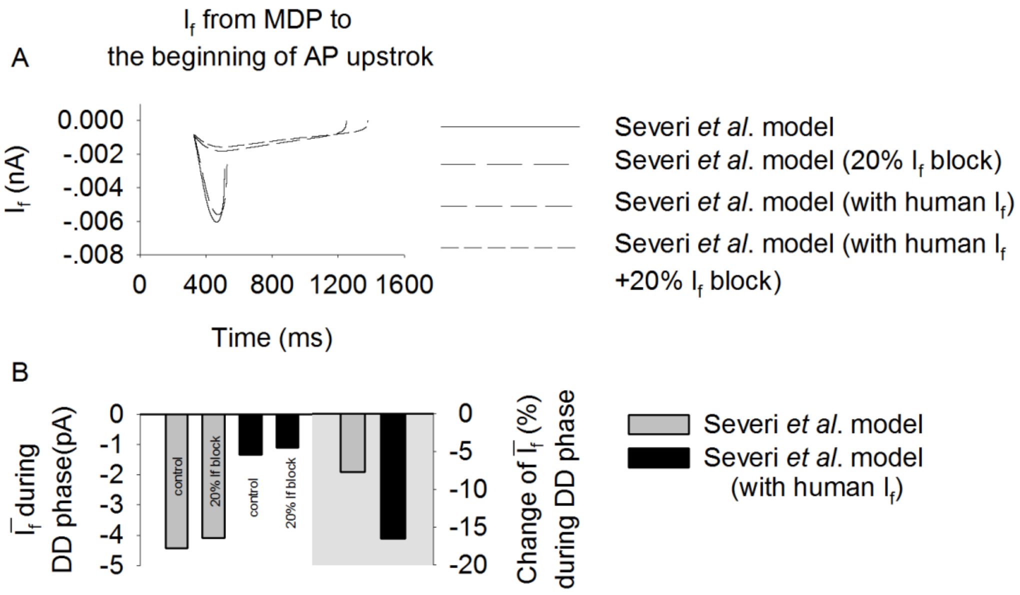

Supplementary Figure S2. Simulated I_f during diastolic depolarization phase of the action potential in the rabbit-like and human-like I_f formulations models.
