# Supplementary material for: The Functional Role of Hyperpolarization Activated Current (If) on Cardiac Pacemaking in Human vs. in the Rabbit Sinoatrial Node: A Simulation and Theoretical Study
Source: Front Physiol. 2021 Aug 19;12:582037. doi: 10.3389/fphys.2021.582037 (PMC8417414; doi:10.3389/fphys.2021.582037)
Supplement: Supplementary file 3 [file Image_3.pdf]

Supplementary Material

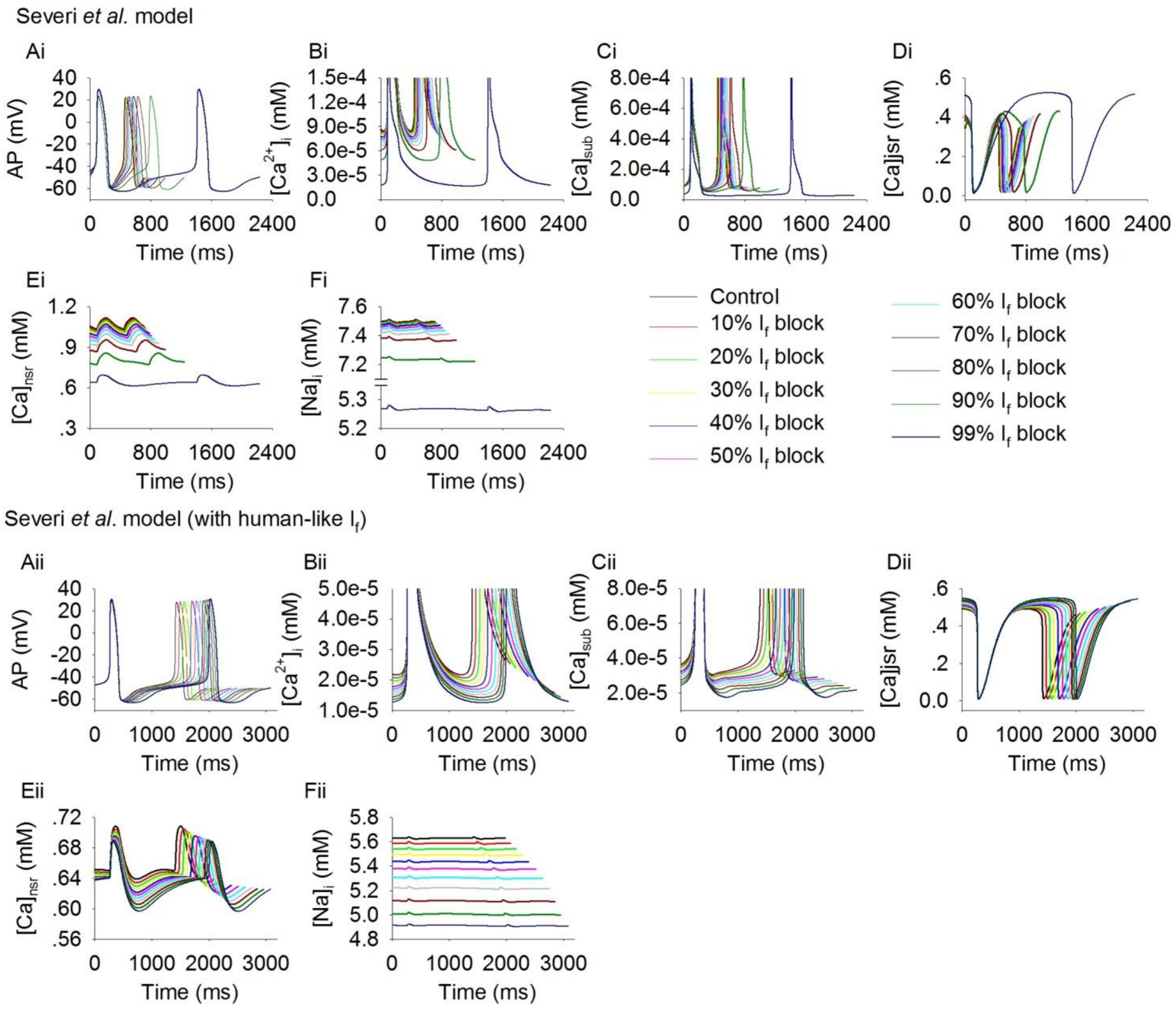

Supplementary Figure S3. Simulated effect of I_f block on the Ca^{2+} transient in rabbit-like (Ai-Ei) and human-like (Aii-Eii) SAN cell models. Ai,Aii: the action potentials; Bi,Bii: Ca^{2+} transient in the myoplasmic space ; Ci,Cii: Ca^{2+} transient in the myoplasmic sub-space; Di,Dii: Ca^{2+} transients in the junctional SR; Ei,Eii: Ca^{2+} transient in the network SR space; Fi,Fii: intracellular Na^+ concentration.
