# Supplementary material for: The Functional Role of Hyperpolarization Activated Current (If) on Cardiac Pacemaking in Human vs. in the Rabbit Sinoatrial Node: A Simulation and Theoretical Study
Source: Front Physiol. 2021 Aug 19;12:582037. doi: 10.3389/fphys.2021.582037 (PMC8417414; doi:10.3389/fphys.2021.582037)
Supplement: Supplementary file 4 [file Image_4.pdf]

Supplementary Material

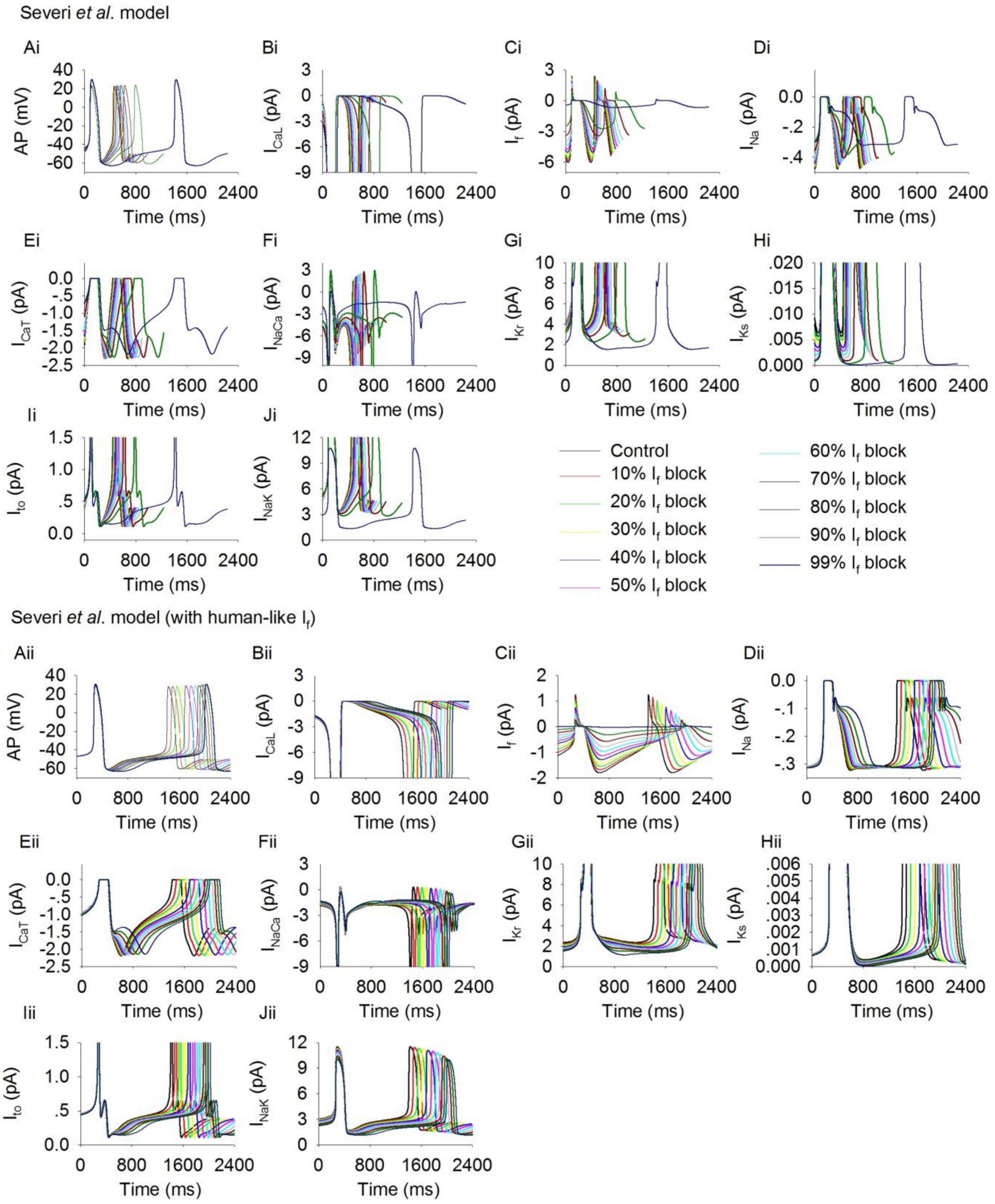

Supplementary Figure S4. Simulated *I*f block effects on spontaneous action potentials and underlying currents by the Severi *et al*. model with rabbit *I*f (Ai-Ji) and human *I*f (Aii-Jii) formulations.
