# Supplementary material for: The Functional Role of Hyperpolarization Activated Current (If) on Cardiac Pacemaking in Human vs. in the Rabbit Sinoatrial Node: A Simulation and Theoretical Study
Source: Front Physiol. 2021 Aug 19;12:582037. doi: 10.3389/fphys.2021.582037 (PMC8417414; doi:10.3389/fphys.2021.582037)
Supplement: Supplementary file 5 [file Image_5.pdf]

# Supplementary Material

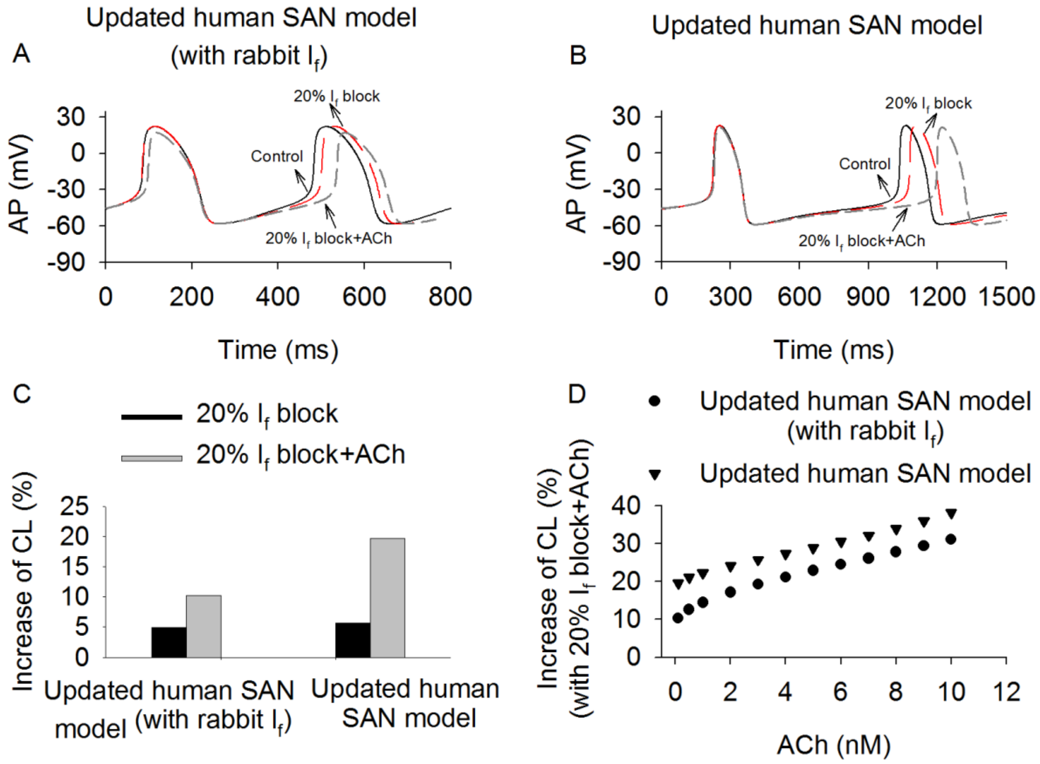


**Supplementary Figure S5.** Acetylcholine effct on sponteneous action potentials under control and 20% $I_f$ block conditions in the human sinus node model with rabbit-like and human-like $I_f$ formulations. A,B: the action potentials; C: the increase of pacemaking CL in the conditions of 20% $I_f$ block alone and cimbined with ACh (0.1 nM) action; D: combined effect of ACh (from 0.1 nM to 10 nM) and 20% $I_f$ block on pacemaking CL in the rabbit-like and human-like $I_f$ formulations models.
