# Supplementary material for: The Functional Role of Hyperpolarization Activated Current (If) on Cardiac Pacemaking in Human vs. in the Rabbit Sinoatrial Node: A Simulation and Theoretical Study
Source: Front Physiol. 2021 Aug 19;12:582037. doi: 10.3389/fphys.2021.582037 (PMC8417414; doi:10.3389/fphys.2021.582037)
Supplement: Supplementary file 6 [file Image_6.pdf]

Supplementary Material

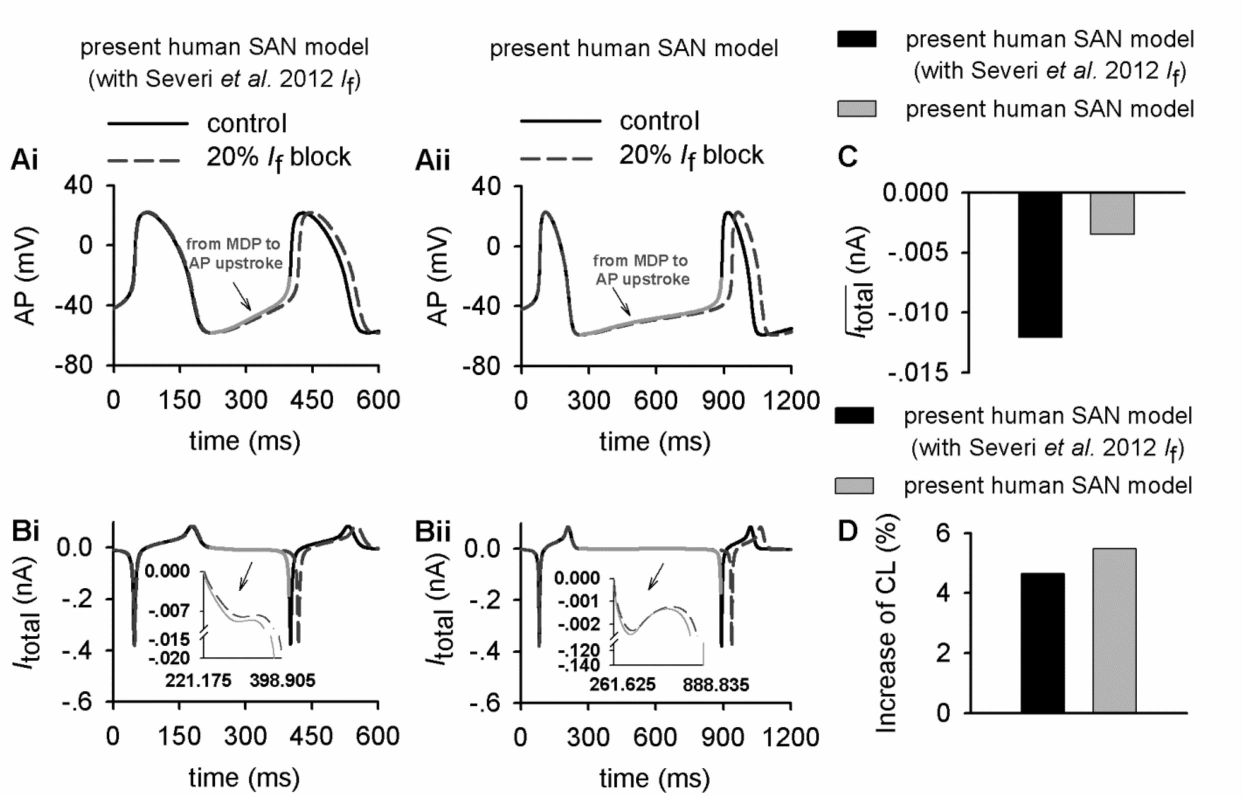

Supplementary Figure S6. Simulated the inverse relationship of I_{total} and the increasing of CL in control (solid lines) and 20% I_f reduction (dotted lines) to validate theoretical analysis result (equation (2)) in method section (*p*=0.2, modeling 20% I_f reduction). Ai:Aii: time course of action potentials for human SAN model with rabbit-like and human-like I_f formulations respectively (grey lines represents diastolic depolarization voltage changes from MDP to the voltage at the beginning of AP upstroke in control condition); Bi:Bii: time courses of I_{total} (grey lines represents diastolic depolarization I_{total} changes from MDP to the voltage at the beginning of AP upstroke in control condition). C: averaged value of I_{total} amplitude during the diastolic depolarization phase for the rabbit-like (black) and present human-like (grey) I_f formulations models; D: the increase of CL in the two models.
