# Supplementary material for: The Functional Role of Hyperpolarization Activated Current (If) on Cardiac Pacemaking in Human vs. in the Rabbit Sinoatrial Node: A Simulation and Theoretical Study
Source: Front Physiol. 2021 Aug 19;12:582037. doi: 10.3389/fphys.2021.582037 (PMC8417414; doi:10.3389/fphys.2021.582037)
Supplement: Supplementary file 7 [file Image_7.pdf]

Supplementary Material

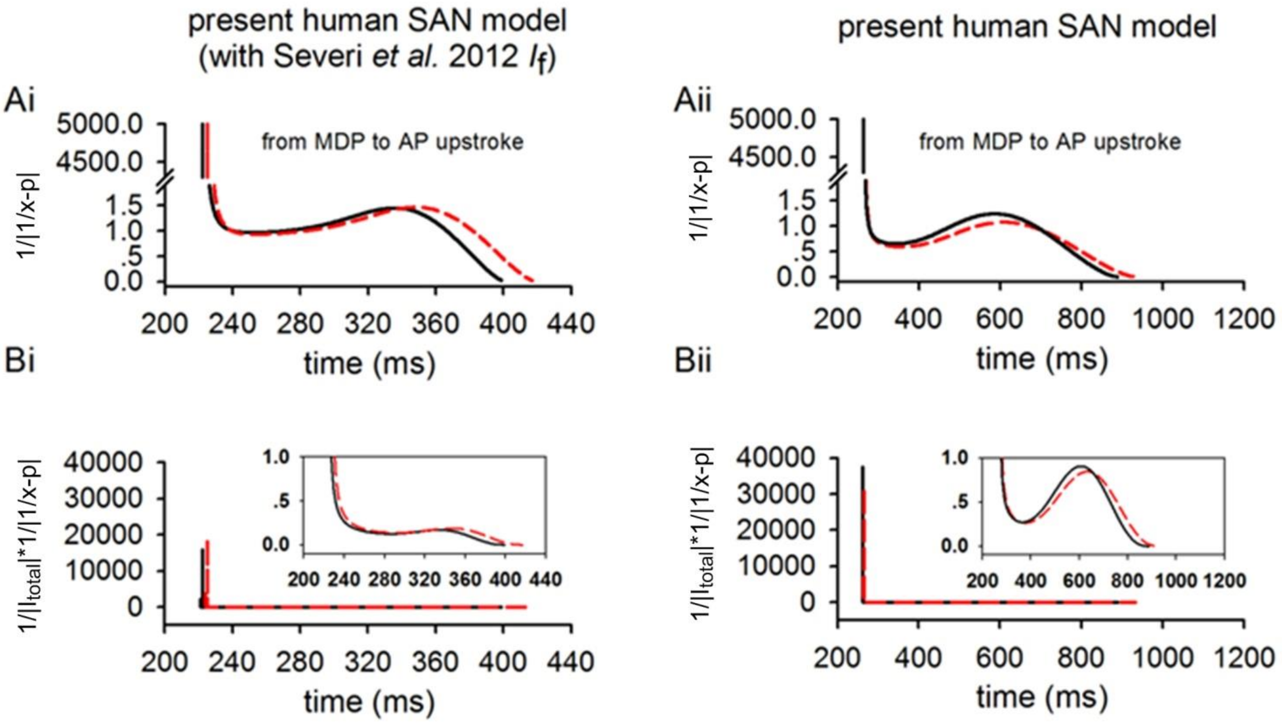

Supplementary Figure S7. Computed values of $\frac{1}{|1/x-p|}$ and $\frac{1}{|I_{total}|}\cdot\frac{1}{|1/x-p|}$ to validate theoretical analysis result (equation (2)) in method section (p=0.2, modeling 20% I_f reduction). The highlighted lines shown in this figure are during the diastolic depolarization phase of the action potentials of control (full line) and 20% I_f reduction (dotted line) cases for the human sinus node model with rabbit-like (Ai,Bi) and human-like (Aii,Bii) I_f formulation.
