# Supplementary material for: The Functional Role of Hyperpolarization Activated Current (If) on Cardiac Pacemaking in Human vs. in the Rabbit Sinoatrial Node: A Simulation and Theoretical Study
Source: Front Physiol. 2021 Aug 19;12:582037. doi: 10.3389/fphys.2021.582037 (PMC8417414; doi:10.3389/fphys.2021.582037)
Supplement: Supplementary file 8 [file Image_8.pdf]

Supplementary Material

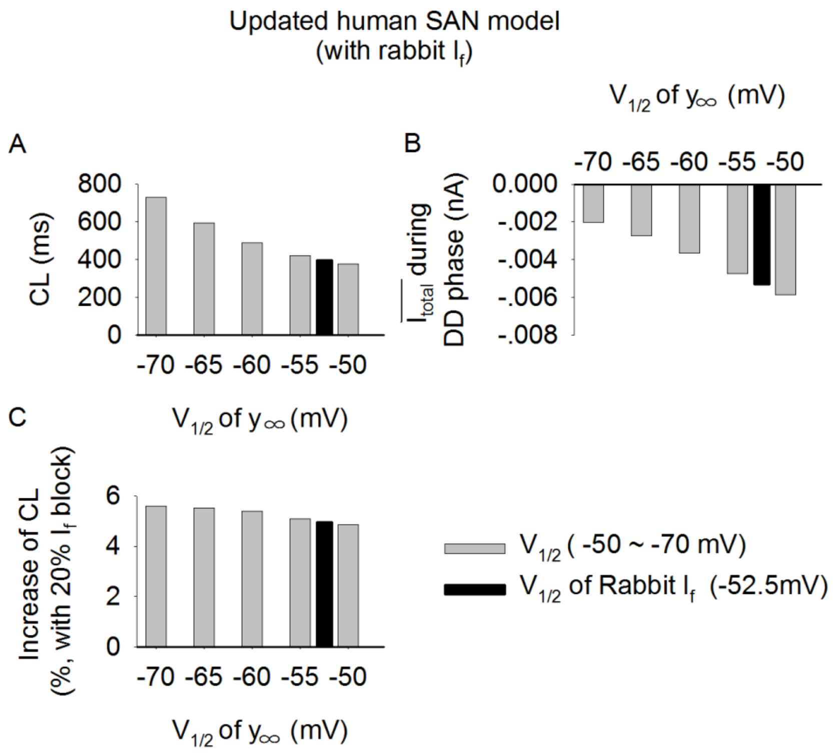

Supplementary Figure S8. Effect of the changes in half-activated voltage ($V_{1/2}$) of the steady state activation variable (y_∞) of I_f on pacemaking CL in present human sinoatrial node model with rabbit-like I_f formulation. $V_{1/2}$ changed from -50 mV to -70 mV.
