# Supplementary material for: The Functional Role of Hyperpolarization Activated Current (If) on Cardiac Pacemaking in Human vs. in the Rabbit Sinoatrial Node: A Simulation and Theoretical Study
Source: Front Physiol. 2021 Aug 19;12:582037. doi: 10.3389/fphys.2021.582037 (PMC8417414; doi:10.3389/fphys.2021.582037)
Supplement: Supplementary file 9 [file Table_1.docx]

Supplementary Material

**Supplementary Table S1.** Changes of main action potential parameters in the condition of *I*_f_ block from 0% to 100% in the rabbit-like and human-like SAN models.

|  | Rabbit-like SAN model  (Severi *et al*. model) | | | | | Human-like SAN model  (Severi *et al*. model (with human *I*_f_)) | | | | |
| --- | --- | --- | --- | --- | --- | --- | --- | --- | --- | --- |
| *I*_f_ block  (%) | CL (ms) | PR reduction（%） | MDP  (mV) | APD_90_ (mV) | AP upstroke start voltage  (mV) | CL (ms) | PR  Reduction  （%） | MDP  (mV) | APD_90_ (mV) | AP upstroke start voltage  (mV) |
| 0 | 354.8 | 0 | -58 | 109.7 | -22.6 | 1139.4 | 0 | -60.3 | 127.8 | -22.2 |
| 10 | 362.3 | -2.1 | -58.1 | 109.9 | -22.6 | 1208.6 | -5.8 | -60.5 | 128.3 | -22.2 |
| 20 | 371.1 | -4.4 | -58.2 | 110.1 | -22.6 | 1280.7 | -11.1 | -60.6 | 128.9 | -22.2 |
| 30 | 381.5 | -7.0 | -58.3 | 110.3 | -22.6 | 1354.7 | -16.0 | -60.8 | 129.6 | -22.2 |
| 40 | 394.1 | -10.0 | -58.5 | 110.5 | -22.6 | 1429.6 | -20.4 | -60.9 | 130.4 | -22.2 |
| 50 | 410.0 | -13.5 | -58.6 | 110.6 | -22.6 | 1504.1 | -24.3 | -61.1 | 131.3 | -22.1 |
| 60 | 431.3 | -17.7 | -58.8 | 110.7 | -22.6 | 1575.0 | -27.7 | -61.4 | 132.4 | -22.1 |
| 70 | 462.7 | -23.3 | -59.1 | 110.7 | -22.6 | 1624.4 | -29.9 | -61.7 | 133.8 | -22.1 |
| 80 | 518.4 | -31.6 | -59.7 | 111.5 | -22.5 | 1713.5 | -33.6 | -62.1 | 134.9 | -22.1 |
| 90 | 687.4 | -48.4 | -60.9 | 109.6 | -22.5 | 1724.3 | -34.0 | -62.6 | 136.9 | -22.1 |
| 100 | - | - |  | - | - | 1769.3 | -35.7 | -63.6 | 138.5 | -22.0 |
