# Supplementary material for: The Functional Role of Hyperpolarization Activated Current (If) on Cardiac Pacemaking in Human vs. in the Rabbit Sinoatrial Node: A Simulation and Theoretical Study
Source: Front Physiol. 2021 Aug 19;12:582037. doi: 10.3389/fphys.2021.582037 (PMC8417414; doi:10.3389/fphys.2021.582037)
Supplement: Supplementary file 10 [file Data_Sheet_1.docx]

Supplementary Material

# 1 Update the human sinus node cell model

Fabbri *et al.* ([Fabbri et al., 2017](#_ENREF_2)) developed a human sinus node cell model that closely matches to experimental data of APs properties and calcium transient. However, most equations for the activation/inactivation and dynamic properties of the ionic currents (except *I*_f_, *I*_Kr_ and *I*_Ks_) of the model were inherited from rabbit SAN cell model, and current densities were modified by automatic optimization method based on action potential characteristics, forming some limitations of the model. For example, block of *I*_CaT_ abolished the pacemking activity, whilst data from rabbit SAN cells ([Satoh, 1997](#_ENREF_4)) and human patients ([Madle et al., 2001](#_ENREF_3)) suggested more modest effects (e.g. complete *I*_CaT_ block produced about 15 - 50% ([Satoh, 1997](#_ENREF_4)) or even 88% pacing cycle length prolongation in rabbit SAN cells). Therefore, we updated the model to balance the interaction of currents during the diastolic depolarization phase by:

1. decreasing the permeability of *I*_CaL_ by 20%, and shifted the steady state activation variables of *I*_CaL_ (*L*_∞_) by -1mV (dL_∞_=1/(1+e^-(V+17.45)/4.337)^);
2. decreasing the conductance of *I*_CaT_ (*p*_CaT_) by 33%;
3. increasing the time constant of Ca^2+^ diffusion from the sub-sarcolemmal space to the myoplasm (*τ*_Cadif_) by 11.1%

The updated model was validated by its ability to produce AP characteristics matching to experimental data as shown in supplementary Figure S1.

In supplementary Figure S1, the characteristics of action potential and Ca^2+^ transient properties were computed and validated against experimental data. The parameters of the computed action potentials (supplementary Figure S1A) were in agreement with experimental data from the study of Verkerk *et al*. ([Verkerk et al., 2007](#_ENREF_7)). The Ca^2+^ transient properties were similar to those in the Fabbri *et al*. model. In the updated model, whilst blocking I_CaT_ though prolonged the diastolic depolarization phase, but did not abolish the action potential (increasing pacemaking CL by 96%). Full block of *I*_f_ increased pacemaking CL by 40.6%, which is similar to the results as seen in rabbit sinus node (increase by 19-30% in the study of Denyer and Brown ([Denyer and Brown, 1990](#_ENREF_1)); and by 57% in the study of van Ginneken and Giles ([van Ginneken and Giles, 1991](#_ENREF_6)), of which though is greater than the effect of Cs^+^ (2 mmol/L) as seen in the study of Verkerk *et al*. ([Verkerk et al., 2007](#_ENREF_7))).

## REFERENCES

Denyer, J.C., and Brown, H.F. (1990). Pacemaking in rabbit isolated sino-atrial node cells during Cs^+^ block of the hyperpolarization-activated current *i*_f_. *J Physiol* 429**,** 401-409.

Fabbri, A., Fantini, M., Wilders, R., and Severi, S. (2017). Computational analysis of the human sinus node action potential: model development and effects of mutations. *J Physiol* 595**,** 2365-2396.

Madle, A., Linhartova, K., and Koza, J. (2001). Effects of the T-type calcium channel blockade with oral mibefradil on the electrophysiologic properties of the human heart. *Med Sci Monit* 7**,** 74-77.

Satoh, H. (1997). Electrophysiological actions of ryanodine on single rabbit sinoatrial nodal cells. *Gen Pharmacol* 28**,** 31-38.

Severi, S., Fantini, M., Charawi, L.A., and Difrancesco, D. (2012). An updated computational model of rabbit sinoatrial action potential to investigate the mechanisms of heart rate modulation. *J Physiol* 590**,** 4483-4499.

Van Ginneken, A.C., and Giles, W. (1991). Voltage clamp measurements of the hyperpolarization-activated inward current *I*_f_ in single cells from rabbit sino-atrial node. *J Physiol* 434**,** 57-83.

Verkerk, A.O., Wilders, R., Van Borren, M.M., Peters, R.J., Broekhuis, E., Lam, K., Coronel, R., De Bakker, J.M., and Tan, H.L. (2007). Pacemaker current (*I*_f_) in the human sinoatrial node. *Eur Heart J* 28**,** 2472-2478.
