# Supplementary material for: The Functional Role of Hyperpolarization Activated Current (If) on Cardiac Pacemaking in Human vs. in the Rabbit Sinoatrial Node: A Simulation and Theoretical Study
Source: Front Physiol. 2021 Aug 19;12:582037. doi: 10.3389/fphys.2021.582037 (PMC8417414; doi:10.3389/fphys.2021.582037)
Supplement: Supplementary file 11 [file Data_Sheet_2.docx]

Supplementary Material

# 2 Initial values in the Severi *et al*. model (with human *I*_f_ formulation)

I = 1.41299e-9

O = 1.9980e-8

R= 1.0493

RI = 0.0742

fCMi = 0.0126464

fCMs = 0.01326

fCQ = 0.332049

fTC = 6.03724e-3

fTMC = 0.122082

fTMM = 0.775566

Ca_jsr_ = 0.415099

Ca_nsr_ = 0.639261

Ca_sub_ = 3.19976e-5

Ca_i_ = 3.04074e-5

V_m_ = -52.0114

Na_i_ = 5.63076

dL = 5.20374e-4

fCa = 0.908569

fL = 0.953286

dT = 0.0762193

fT =0 .157737

a= 3.00643e-3

paF = 0.0119265

paS = 0.346624

pi = 0.797585

n = 2.93776e-3

h =0 .0205513

m = .0230745;

y = 0.0138945

q = 0.579941

r = 8.48565e-3
