# Supplementary material for: The Functional Role of Hyperpolarization Activated Current (If) on Cardiac Pacemaking in Human vs. in the Rabbit Sinoatrial Node: A Simulation and Theoretical Study
Source: Front Physiol. 2021 Aug 19;12:582037. doi: 10.3389/fphys.2021.582037 (PMC8417414; doi:10.3389/fphys.2021.582037)
Supplement: Supplementary file 12 [file Data_Sheet_3.docx]

Supplementary Material

# 3 Theoretical analysis validation in present human sinus node cell model

We additionally validated our theoretical analysis based on the human sinus node cell model the human-like *I*_f_ formulation and the rabbit-like *I*_f_ formulation (taken from Severi *et al*. ([Severi et al., 2012](#_ENREF_5)) model). Results were shown in supplementary Figure S5 and Figure S6.
