# Supplementary material for: The Functional Role of Hyperpolarization Activated Current (If) on Cardiac Pacemaking in Human vs. in the Rabbit Sinoatrial Node: A Simulation and Theoretical Study
Source: Front Physiol. 2021 Aug 19;12:582037. doi: 10.3389/fphys.2021.582037 (PMC8417414; doi:10.3389/fphys.2021.582037)
Supplement: Supplementary file 13 [file Data_Sheet_4.docx]

Supplementary Material

# 4 Modelling the effect of ACh

The effect of ACh was simulated by decreasing of *I*_CaL_ and rate constant for Ca^2+^ uptake SERCA pump into network SR (P_up_), activation of ACh sensitive potassium current (*I*_KACh_), and a shift in the steady state activation variable (y_∞_) and time constant (τ_y_) of *I*_f_, with details as follows:

1. Decrease of *I*_CaL_:

$$I_{CaL}^{^{'}}=(1.0-\frac{0.3\cdot ACh}{ACh+0.0009})\cdot I_{CaL}$$

1. Decrease in P_up_:

$$p_{up}^{'}=(1.0-\frac{0.7\cdot ACh}{ACh+0.00009})\cdot p_{up}$$

1. Activation of *I*_KACh_ (formulations):

$$I_{KACh}=\left\{\begin{matrix} g_{KACh}\cdot(V-E{}_{K})\cdot(1+e^{\frac{V+20}{20}})\cdot a,ifACh>0 \\ \begin{matrix} & & \end{matrix}0,otherwise \end{matrix}\right.$$

$$a_{\infty}\text{=}\frac{a_{a}}{a_{a}\text{+}\beta_{a}}$$

$$a_{a}\text{=}\frac{\text{3.5988-0.0256}}{\text{1+}\frac{0.0000012155}{ACh^{1.6951}}}+0.0256$$

$$\beta_{a}\text{=10}\cdot e^{0.0133\cdot(V+40)}$$

$$\tau_{a}\text{=}\frac{1}{a_{a}\text{+}\beta_{a}}$$

$$\frac{\text{d}a}{\text{d}t}\text{=}\frac{a_{\infty}-a}{\tau_{a}}$$

Simulations used g_K,ACh_ values the same as used in the Severi *et al.* ([Severi et al., 2012](#_ENREF_55)) and Fabbri *et al*. model([Fabbri et al., 2017](#_ENREF_30)). For the human-like model (Severi model with human-like *I*_f_), we used the same g_K,ACh_ as used in the human SAN model published by Fabbri *et al* 2017 (6.0526×10^-5^μS/pF). For the rabbit-like model (Fabbri model with rabbit-like *I*_f_ in the Online Supplement), we use the same g_K,ACh_ as used in the rabbit SAN model published by Severi *et al*. 2012 (2.7×10^-4^μS/pF).

1. ACh effect on *I*_f_: the steady state activation variable (y_∞_) and time constant (τ_y_) of *I*_f_ was shifted by *k* (unit: mV), which takes the form:

$$k=\text{-1.0-9.898}\cdot\frac{\text{ACh}^{0.618}}{\text{ACh}^{0.618}+0.00122423}$$
